# Supplementary material for: Training tactile sensors to learn force sensing from each other
Source: Nat Commun. 2026 Jan 28;17:2101. doi: 10.1038/s41467-026-68753-1 (PMC12953886; doi:10.1038/s41467-026-68753-1)
Supplement: Supplementary file 1 — Supplementary Information [file 41467_2026_68753_MOESM1_ESM.pdf]

Supplementary Information

Training Tactile Sensors to Learn Force Sensing from Each Other

Zhuo Chen[1*], Ni Ou[1], Xuyang Zhang[1], Zhiyuan Wu[1], Yongqiang Zhao[1], Yupeng Wang[1], Emmanouil Spyrakos Papastavridis[1], Nathan Lepora[2], Lorenzo Jamone[3], Jiankang Deng[4*], Shan Luo[1*]

[1]King's College London, London, United Kingdom.
[2]University of Bristol, Bristol, United Kingdom.
[3]University College London, London, United Kingdom.
[4]Imperial College London, London, United Kingdom.

*Corresponding authors. Email:
zhuo.7.chen@kcl.ac.uk, j.deng16@imperial.ac.uk, shan.luo@kcl.ac.uk

This PDF file includes:

Supplementary Text 1 to 10

Supplementary Figure 1 to 45

Supplementary Table 1 to 3

Text 1. Binary marker images as unified tactile representation

Among tactile modality such as marker coordinates, marker images, depth map and 3D point cloud, we choose binary marker images as unified representation by considering the efficiency, robustness and common features among tactile sensors as follow:

1. **Efficiency:** In our work, the whole process of marker conversion and force prediction is fast in all sensors, which can be run in real-time around 29.6 Hz (the camera is 30fps) and demonstrated in our real robot applications with force control (see Supplementary Video 3-7). However, the marker coordinates normally need one more operation following marker segmentation in vision-based tactile sensors. They are extracted based on the marker images by finding the marker centroid with 2D displacement (X axis and Y axis, without information in Z axis). This process also loses information such as marker deformation compared with marker images, which provide essential information for the normal force. Depth map and 3D point clouds is normally used in vision-based tactile sensors calculated by calibration method[1] and neural networks (such as GelSight-mini), thus are more complex and less efficient (higher computational cost) than 2D marker images.
2. **Robustness:** Marker representations are 2D black-white images with binary format (0, 255). This data format is friendly to model training compared with RGB images ranging from 0-255 without considering image background and contact geometry. The npy saving format is uncompressed while only takes 39KB by packing into binary bit (see Supplementary Fig. 37). However, the jpg file takes 57KB (30%+ memory cost than binary image) and introduces ringing and nonzero values near edges due to its compressed format. The CNN model is also robust to missing markers, irregular markers and random noises we introduced in our dataset (see Supplementary Fig. 34). However, marker coordinates are easy to lose track due to the missing/overlapped markers under large deformation (see Supplementary Fig. 35) or artifacts appearing on the image. The missing coordinate is unfriendly to model design and training. While depth map/3D point cloud are hard to extract from sensors with markers (see Supplementary Fig. 36).
3. **Common features among tactile sensors:** Binary marker images can be unified and easy to be converted from both vision-based tactile sensors or non-vision based tactile sensors. For vision-based tactile sensors, we can convert it from markerless tactile images by using regressive network (see Supplementary Fig. 38) or segment from tactile images with physical markers (see Supplementary Fig. 4 and Supplementary Fig. 11). For non-vision based tactile sensors, the marker images can be converted from multichannel signals regardless of three-axis deformation sensing or pressure-only sensing in each taxel (see Supplementary Fig. 11). However, the marker

coordinates are hard to be obtained in non-vision based tactile sensors, such as electronic sensor arrays with pressure-only sensing. For 3D point cloud/depth map, it is hard to generalize in sensor arrays with sparse distributions.

Text 2. Marker-to-marker translation model

The M2M model consists of two main components: a marker encoder-decoder and an image-conditioned diffusion model. As shown in Fig. 2C, the marker encoder transforms deformed images $I_t^{S_i}$ from sensor i and the reference image $I_0^{T_j}$ from sensor j into latent vectors $z_t^{S_i}$ and $z_0^{T_j}$ respectively, while the marker decoder converts the output latent vector $z_t^{G_i}$ from the diffusion model to the generated deformed images $I_t^{G_i}$. The image-conditioned diffusion model fuses latent vector $z_t^{S_i}$ with the conditional input $z_0^{T_j}$ through cross-attention mechanisms[49] and denoises the fused feature map to produce latent vectors $z_t^{G_i}$. This end-to-end architecture enables direct translation of marker-based images from $I_t^{S_i}$ to $I_t^{G_i}$ with the image style of $I_t^{T_j}$ while preserving the deformation from $I_t^{S_i}$. The training objective combines two primary components to train the model in a pixel-to-pixel manner[2], i.e. an adversarial loss L_{gan}[3], and a reconstruction loss L_{rec} incorporating L2 and LPIPS[4] loss.

Adversarial Loss The adversarial loss aims to align the distribution of generated tactile images $p(I^G)$ with the target images $p(I^T)$. The discriminator D_T learns to differentiate between generated images I^G and real target images I^T. The adversarial loss is formulated as:

$$\mathrm{L}_{gan} = \mathrm{E}_{I^T \sim p(I^T)}[\log D_T(I^T)] + \mathrm{E}_{I^S \sim p(I^S)}[\log(1 - D_T(G(I^S, I_0^T))] \quad (1)$$

where G minimizes this objective while D_T maximizes it: $\min_G \max_{D_T} \mathrm{L}_{gan}$.

Reconstruction Loss The reconstruction loss L_{rec} ensures both pixel-level and perceptual-level similarity between generated images I^{G_i} and target images I^{T_j} through L2 and LPIPS metrics, capturing subtle marker displacement during translation:

$$\mathrm{L}_{rec} = \sum_{i=1}^{n}\sum_{j=1}^{m} \lambda_{\mathrm{L2}} \mathrm{E}_{I^{S_i} \sim p(I^{S_i})} \left\| I^{T_j}, G(I^{S_i}, I_0^{T_j}) \right\|_2 + \lambda_{\mathrm{Lpips}} \mathrm{E}_{I^{S_i} \sim p(I^{S_i})} \left\| I^{T_j}, G(I^{S_i}, I_0^{T_j}) \right\|_{PIPS} \quad (2)$$

Where λ_{L2} is the weight for L2 loss, λ_{Lpips} is the weight for LPIPS loss.

Overall Objective The complete learning objective for the generative model combines the above losses with weights λ_{gan} and λ_{rec}:

$$\arg\min \lambda_{gan} \mathrm{L}_{gan} + \lambda_{rec} \mathrm{L}_{rec} \tag{3}$$

Marker encoder-decoder. As shown in Supplementary Fig. 28, we adapt the variational autoencoder (VAE) architecture from SD-Turbo[5]. The VAE processes marker images with a size of 256×256 and employs an encoder-decoder structure: an encoder that compresses marker patterns into a latent space, and a decoder that reconstructs marker patterns from these latent representations. To optimize the model's performance while maintaining parameter efficiency, we implement Low-Rank Adaptation (LoRA)[6] for efficient fine-tuning. The LoRA is with rank-4 adaptation on key network components, including convolutional layers and attention modules. The training objective combines reconstruction loss (L1 and L2) with a KL divergence loss to balance accurate pattern reconstruction with latent space regularization. This architecture enables effective compression of marker patterns into a structured latent space while preserving essential geometric and spatial relationships between different marker types.

Image-conditioned diffusion model. The conditional diffusion model is based on the UNet[7] architecture from SD-Turbo (Supplementary Fig. 28) combined with a DDPM Scheduler[8]. We implement a one-step diffusion process[2] for efficient marker pattern translation. The UNet model is also augmented with LoRA adaptation (rank-8) applied to key network components, including attention layers, convolutional layers, and projection layers. We split the reconstruction loss L_{rec} into $\mathrm{L}_{\mathrm{Lpips}}$ and L_{L2}. The model was optimized using a multi-component loss function:

$$\mathrm{L} = \lambda_{\mathrm{gan}} \mathrm{L}_{\mathrm{gan}} + \lambda_{\mathrm{Lpips}} \mathrm{L}_{\mathrm{Lpips}} + \lambda_{\mathrm{L2}} \mathrm{L}_{\mathrm{L2}} \tag{4}$$

where $\lambda_{\mathrm{gan}} = 0.5$, $\lambda_{\mathrm{Lpips}} = 5.0$, and $\lambda_{\mathrm{L2}} = 1.0$ to balance the contributions of adversarial, LPIPS, and L2 loss respectively. We employed a CLIP-based vision-aided discriminator[9] with multilevel sigmoid loss for the adversarial component, and a VGG-based LPIPS network[4] for perceptual loss computation.

Pretraining for the marker encoder-decoder. The marker encoder-decoder is first trained on the simulation dataset for marker feature extraction. All raw marker images are 640×480 pixels with packed bits file in npy format (see Supplementary Fig. 37). We employ an 80-20 train-test split. All images are preprocessed to a uniform size of 256×256 pixels and normalized to [0,1] range. The model is trained using AdamW optimizer with a learning rate of 1×10^{-4}, betas=(0.9, 0.999), and weight decay of 1×10^{-2}. We employ mixed-precision training (FP16) with a batch size of 4. The loss function combined a reconstruction loss (L1 + L2) and KL divergence with weights of 1.0 and 1×10^{-6} respectively. Training

proceeded for 100,000 steps. The training process for the marker encoder-decoder is demonstrated in Supplementary Fig. 30.

Pretraining for M2M model with simulation data. We load the pretrained marker encoder-decoder for the M2M model. For the encoder for the image condition, we freeze the weights to ensure the extracted features are fixed. The training process utilizes all the 132 combinations from the simulation dataset with 80-20 train-test split. Each training sample in one batch consists of a triplet: a deformed marker image $I_t^{S_i}$ from sensor i, its corresponding paired marker image $I_t^{T_j}$ from sensor j, and a reference marker image $I_0^{T_j}$ from sensor j. The model is trained using AdamW optimizer with an initial learning rate of 5×10^{-6} with 500 warm-up steps, betas = (0.9, 0.999), epsilon=1×10^{-8}, and weight decay of 1×10^{-2}. Training proceeded with a batch size of 4. The training process is shown in Supplementary Fig. 31.

Training for M2M model with real-world data. For the homogeneous translation, we first split the homogeneous location-paired image data into two groups with seen objects and unseen objects. We finetune the simulation pretrained model using the seen group with an 80-20 train-test split with the same hyperparameters as above for the simulation data. The training process for the homogeneous translation is shown in Supplementary Fig. 32. The training for the material effect data uses the same process and hyperparameters but involves loading the model trained with homogeneous data as the pretrained model. The training for the heterogeneous data loads the model weights trained with homogeneous data. The hyperparameters are the same as the homogeneous training except we change the batch size to 16 for speeding up training. The training process for the heterogeneous translation is shown in Supplementary Fig. 33.

Inference process. For model inference, we utilize the mean vector, without variance, of the latent distribution from the marker encoder to ensure deterministic outputs. For datasets in homogeneous translation, material effect and heterogeneous translation, each one is preprocessed using consistent image transformations, including resizing and normalization. The model processes images in batches of 8, generating images with a size of 256×256 that are subsequently upscaled to the target resolution (640×480) using Lanczos interpolation. The upscaled outputs are then thresholded to binary marker images. The results are saved as compressed binary Numpy arrays.

Text 3. Spatiotemporal force prediction model

Model Architecture. The model consists of four main components demonstrated in Supplementary Fig. 29: a marker feature encoder backbone, a spatiotemporal module with convolutional GRU (ConvGRU)[10],

a post-processing network with ResNet Unit, and a regression head with multilayer perceptron (MLP). The input to our model is a sequence of tactile images with shape S×N×3×256×256, where S is the sequence length, N is the batch size, and each image has 3 channels with 256×256 spatial resolution. The marker feature encoder processes these images through three convolutional blocks, each incorporating instance normalization and dropout. The first block reduces spatial dimensions to 128×128 while increasing channels to 64, the second block further reduces to 64×64 with 96 channels, and the third block outputs features at 32×32 resolution with 128 channels. These spatial features are then processed by a ConvGRU module that maintains the 32×32 spatial resolution while capturing temporal dependencies across the sequence. With a hidden state dimension of 128 channels, the ConvGRU tracks temporal patterns while preserving spatial information. The temporal features undergo spatial dimension reduction through two residual blocks (stride 2), expanding the channel dimension from 128 to 256, then to 512, while reducing spatial dimensions to 16×16 and 8×8 respectively. An adaptive average pooling layer collapses the remaining spatial dimensions to 1×1, producing a 512-dimensional feature vector per timestep. The regression head maps these features to three-axis force predictions using a fully connected layer followed by sigmoid activation. This architecture effectively combines spatial and temporal processing to capture both the detailed marker deformations in individual frames and their evolution over time, enabling accurate prediction of three-axis force from tactile image sequences.

The network is optimized using a mean absolute error (MAE) loss function:

$$\mathrm{L}_{\mathrm{MAE}} = \frac{1}{N}\sum_{i=1}^{N} || \hat{F}_i - F_i ||_1 \tag{5}$$

where $\hat{F}_i$ and F_i denote the predicted and ground-truth forces respectively

Model Training. The image data undergoes preprocessing including resizing to 256×256 pixels and normalization using ImageNet statistics (mean=[0.485, 0.456, 0.406], std=[0.229, 0.224, 0.225]). Force measurements are normalized using pre-computed global minimum and maximum values to ensure consistent scaling across different samples. Our dataloader implements dynamic sequence sampling, where for each batch, we randomly sample sequence lengths between the first frame and the maximum available length with at least two frames, enabling the model to learn from varying temporal contexts. For model initialization, we employ normalization for convolutional layers and constant initialization for normalization layers. The training process follows a two-stage approach: first, we pre-train the model on a single randomly selected sensor with a learning rate of 0.1 for 40 epochs, then fine-tune on the complete dataset with a learning rate of 1×10^{-3} for another 40 epochs. We use SGD optimization with momentum

(0.9) and weight decay 5×10^{-4}, along with a learning rate scheduler. During training, we utilize a custom collate function that handles varying sequence lengths through dynamic padding, where shorter sequences are padded to match the batch's sampled sequence length by repeating the last frame. The model is trained with a batch size of 4 using L1 loss between predicted and ground truth forces, exclusively on the seen group data, with early stopping based on validation performance.

Model Inference. During inference, our model processes tactile image sequences to predict three-axis force. The inference pipeline utilizes a modified data loading scheme where, unlike training, we process the complete sequence length without random sampling. The dataloader maintains the same image preprocessing pipeline (resizing to 256×256 and normalization with ImageNet statistics). For both source and target domain evaluation, we load full sequences with a batch size of 1 to ensure consistent temporal processing across all samples. The predictions undergo denormalization using globally tracked minimum and maximum force values the same as in training to restore the actual force scale. We evaluate the model's performance using multiple metrics: Mean Absolute Error (MAE) for individual force components (F_x, F_y, F_z), MAE for total force magnitude F_t, and R^2 values to assess prediction accuracy over the whole force range. Notably, while our unsupervised method has shown impressive performance, a gap remains compared to supervised learning approaches. Enhancing accuracy may involve compensating for additional material properties such as Poisson's ratio, roughness, and viscosity. Alternatively, few-shot finetuning using force labels from simple gauges, weighted objects, or calibrated tactile sensors could help close this gap.

Text 4. Ablation study

For marker-to-marker translation model, we firstly introduce a baseline, cycleGAN[11], as it is bi-directional translation compared with the pixel2pixel GAN with only unidirectional translation. However, we want to highlight cycleGAN can only deal with the translation between two sensors, which is not able to transfer across more than two sensors within one model. We test its performance by using both RGB images and binary marker images. The models are trained in 100,000 iterations with the parameters in [11]. As shown in Supplementary Fig. 39A, the model shows inferiority in correctly translating the illumination direction, the orientation of the indenters and the marker patterns by using RGB image. On the other hand, when only use marker images (see Supplementary Fig. 39B), the predicted image did change compared with the source image or the model is hard to converge when transferring from grid pattern to circular pattern. This explains why we did not choose RGB images as the unified modality due to its more complex illumination properties and contact geometry, which also does not exist in electronic sensor arrays. The marker image is binary without background noise, but it

is hard to train with cycleGAN due to its unpaired dataset setting. The model tries to learn the major image style information, such as the illumination or contact geometry information in the RGB images but it focuses on the black area in the marker images, which is hard to capture the marker deformation. While our models capture both adversarial loss and pixel-level loss with L_{gan} and L_{rec} respectively and has been testified to performs well in our original manuscript.

Regarding the model architecture, we firstly examine the role of two primary components proposed in our training objective, i.e. adversarial loss L_{gan} and reconstruction loss L_{rec} (L2 and LPIPS). Three models with L_{gan}, L_{rec} and $L_{gan} + L_{rec}$ respectively are trained in the sim dataset with most varied marker pattern and translation directions. Specifically, we use the weights $\lambda_{\mathrm{gan}} = 0.5$, $\lambda_{\mathrm{Lpips}} = 5.0$, and $\lambda_{\mathrm{L2}} = 1.0$ from our original manuscript for model with $L_{gan} + L_{rec}$, but set $\lambda_{\mathrm{gan}} = 0$ for model only with loss L_{rec} while set $\lambda_{\mathrm{Lpips}} = 0$, $\lambda_{\mathrm{L2}} = 0$ for model only with L_{gan}. All models are trained with 5 epochs. We show the loss curves and the marker-to-marker translation performance in Supplementary Fig. 40. We find that models with L_{gan} only and L_{rec} only cannot predict correct target images, either with artefacts or gray masks. While models with $L_{gan} + L_{rec}$ shows good performance and can predict visual-similar target images without artefacts or gray mask observed in the generated image. The L_{rec} part also speeds up the convergence of L_{gan} in the model with $L_{gan} + L_{rec}$.

Then, we examined the contribution of the pretrained model with simulated dataset. We compare the performance of our model when training with heterogeneous translation dataset with or without (w/o) pretrained model. We use the weights $\lambda_{\mathrm{gan}} = 0.5$, $\lambda_{\mathrm{Lpips}} = 5.0$, and $\lambda_{\mathrm{L2}} = 1.0$ from our original manuscript and train both models with 5 epochs. As shown in Supplementary Fig. 41, the model without pretraining cannot converge and predict artefacts. While the model pretrained with simulated dataset show distinct convergence speed and can transfer from a low-resolution sensor (uSkin, 3-axis) to a high-resolution sensor (TacTip, palm) in a visual-similar pattern. This verifies the contribution of our simulated dataset and necessary of the pretraining stage.

For the force prediction part, we include two baseline models which are commonly used in tactile sensing community, i.e the resnet backbone and renest backbone with additional LSTM module. For our model, we compare the performance of the feature encoder, and feature encoder followed with a convGRU module with above two baselines. The outputs from those four modules are then connected with post-

processing part and a regression head to output three-axis forces. Four models are test with three heterogeneous sensors. For Supplementary Fig. 42, four models are trained with 18 indenters to show the training performance. For Supplementary Fig. 43, 12 indenters (seen group) are used in the training stage while 6 indenters (unseen group) are used in the test stage. All models are firstly trained with 20 epochs with learning with 0.1 and another 20 epochs with learning rate of 0.001. The experiments are calculated with the mean and std trained in three times with three random seed {0,10,20}. The experimental results show that our model shows the lowest force prediction in normal force and shear forces for all sensors.

We also discover that the training batch size (1, 2, 4) affect the performance of our model performance. The experimental results in Supplementary Fig. 44 shows that training with batch size with 1 show twice times larger force errors than models training more than 1. For our model, we normally train the model with batch size larger than 2. In addition, with the increase of trained indenters (see Supplementary Table 1), the model performance increases accordingly as it learns more information from increased data.

Text 5. Trajectory for marker deformation simulation

The trajectory shown Supplementary Fig. 1B covers a grid of contact locations with horizontal steps Δx and Δy of 4 mm and vertical increments Δz of 0.3 mm, reaching a maximum indentation depth $z_{\max}$ of 1.5 mm. This approach yields 45 target contact locations (5 steps in depth $\times 9$ grid) per indenter, resulting in 810 unique deformed meshes in total. For each movement to target location, the indenter is initialized at a position where its bottom surface is parallel to and 10 mm above the elastomer surface. To ensure we are obtaining smooth mesh, we set the world step time to 1×10^{-4} s and the contact speed to -10 mm/s.

Text 6. Fabrication of soft skins

The fabrication process is demonstrated in Supplementary Fig. 2A. First, we mix XPA-565 silicone base (B) with activator (A) using different ratios to control the softness. For homogeneous translation and heterogeneous translation, we use a ratio of 15:1. In material compensation, we employ seven different ratios ranging from 6:1 to 18:1, where higher ratios produce softer elastomers. We pour the mixture into a mold for 4 mm thickness for 24-hour natural curing to obtain transparent silicone elastomer. Next, we print designed markers (see Supplementary Fig. 4A) on sticker paper using an inkjet printer and transfer them onto the cured elastomer. We then prepare a coating mixture by combining aluminum powder and

silver bullet powder with solvent in a 1:1:2.5 ratio, then mix this with silicone elastomer (15:1 ratio) to pour onto the elastomers with markers. The pigment mixture ensures opaqueness while maintaining negligible increase in the elastomers' thickness. After another 24 hours of curing, we cut the elastomer to 20×20 mm dimensions for testing. Notably, increasing the XPA-565 ratio extends the required curing time.

Text 7. Parameters for data collection in real world

For homogeneous and material compensation tests, we implement the following parameters to the parameters defined in Supplementary Fig. 2B: horizontal moving distances $\Delta x = 3$ mm, $\Delta y = 4$ mm, depth step $\Delta z = 0.3$ mm with maximum depth $z_{\max} = 1.2$mm, moving angle $\theta = 30^{\circ}$, and shear distance $\Delta r = 1$mm. This configuration yields $5 \times 4 \times 12 = 240$ target points with varying moving directions and locations.

To reduce the data amount, the heterogeneous translation employs a moving angle $\theta = 45^{\circ}$ with depth parameters of $\Delta z = 0.25$ mm and $z_{\max} = 1$mm for GelSight and uSkin. The parameters for TacTip are configured with $\Delta x = \Delta y = 6.5$ mm, $\Delta z = 1.125$ mm, $z_{\max} = 4.5$ mm, $\theta = 30^{\circ}$ and $\Delta r = 1.5$ mm. This configuration yields $5 \times 4 \times 8 = 160$ target points. This configuration enables image collection at 0.25 mm intervals for GelSight and uSkin to pair with TacTip collected at 1.125 mm intervals, ensuring comparable force ranges collected from TacTip.

Text 8. Parameters of marker conversion for uSkin

Through grid search for the parameters shown in Supplementary Fig. 12A, we determine the optimal visualization parameters: $D_{\min} = 300$, $D_{\max} = 6000$, $\Delta X_{\max} = \Delta Y_{\max} = 0.6$, $S_D = 0.2$, $S_x = S_y = 0.002$. These parameters provide an optimal balance between sensitivity to subtle deformations and clear visualization of larger forces while preventing marker overlap or grid distortion.

Text 9. Material Compensation

9.1 Relationship of Normal Force and Elastic Modulus

According to contact mechanics, when a flat rigid indenter applies normal force F_{normal} in the z direction on an elastic specimen's surface[12], the relationship between force F_{normal} and indentation depth d_z is given by:

$$F_{normal} = \alpha E^* d_z \tag{6}$$

where α is a geometric constant specific to the indenter, and E^* represents effective modulus, i.e. combined modulus of the indenter and the specimen.

For multi-material contacts, the effective modulus satisfies,

$$\frac{1}{E^*} = \frac{1-\nu^2}{E} + \frac{1-\nu'^2}{E'}, \qquad \frac{1}{G^*} = \frac{2-\nu}{4G} + \frac{2-\nu'}{4G'}, \qquad G = \frac{E}{2(1+\nu)} \tag{7}$$

Where E' and ν', and E and ν, describe the elastic modulus and Poisson's ratio of the indenter and the specimen respectively. In the context of contact of a rigid indenter with a soft elastomer, the effective modulus E^* and G^* are determined by the elastomer E and G [12].

Based on Equation (6), we can derive that in a fixed contact depth:

$$F_{normal} \propto E \tag{8}$$

9.2 Relationship of Shear Force and Elastic Modulus

When considering contacts which are loaded both in the normal direction z and in the tangential direction x and y, it is the "Cattaneo–Mindlin problem" [13]. We consider the solution of the tangential contact problem using the Method of Dimensionality Reduction (MDR)[13].The shear force F_{shear} is described by a rigid lateral shift $u^{(0)}$ of the rigid indenter over an elastic base characterized by effective modulus E^* (normal) and G^* (shear). The contact has outer radius a; the inner stick region extends to radius c and the outer annulus ($c < x < a$) slips region.

By MDR superposition, the total shear force is the sum of a stick-core term and a slip-annulus term,

$$F_{shear} = F_{\text{stick}} + F_{\text{slip}}, F_{\text{stick}} = 2cG^* u^{(0)}, F_{\text{slip}} = \mu[F_{normal}(a) - F_{normal}(c)]. \tag{9}$$

Here μ is the Coulomb coefficient of friction and $F_{normal}(a)$ is the normal force associated with a contact of radius a. The MDR problem provides the normal force as

$$F_{normal}(a) = 2E^* \int_0^a \left[d(a) - d(x)\right] dx \tag{10}$$

Where indentation depth $d(x) = g(x)$, $g(x)$ is the shape profile of the indenter in a one-dimensional.

The stick–slip interface is determined by

$$u^{(0)} = \mu \frac{E^*}{G^*}[d(a) - d(c)] \tag{11}$$

which gives the stick–slip boundary with radius c.

Eliminating $u^{(0)}$ from the decomposition using the displacement criterion yields a compact expression for the shear force:

$$\begin{aligned} F_{shear} &= 2cG^* u^{(0)} + \mu[F_{normal}(a) - F_{normal}(c)] \\ &= \mu\{[F_{normal}(a) - F_{normal}(c)] + 2cE^*[d(a) - d(c)]\} \end{aligned} \tag{12}$$

Because $F_{normal}(\cdot) \propto E^*$ *and* $d(\cdot)$ is purely geometric, both bracketed terms in the compact expression are linear in E^*. The shear force approximately satisfies,

$$F_{shear} \propto \gamma E \tag{13}$$

Where γ is the factor related to the difference in geometry g, imposed tangential shift u, modulus ratio E^* / G^* (effective Poisson ratio) and coefficient of friction μ. The effective modulus E^* and G^* are determined by the elastomer E and G similar to normal force.

9.3 Compensation for normal force and shear force

Base above relationship, to compensate the normal force, we can approximately multiply the existing force label F^S_{normal} from the source sensor S with the ratio of modulus to get compensated force label F^{SC}_{normal}

$$F^{SC}_{normal} \approx \frac{E^T}{E^S} F^S_{normal} \tag{14}$$

Regarding shear force F^S_{shear}, the shear force can be compensated:

$$F_{shear}^{SC} \approx \gamma \frac{E^T}{E^S} F_{shear}^{S} \tag{15}$$

where friction weighting factor γ is set as a hyperparameter.

Specifically, the compensation for differences in modulus $\frac{E^T}{E^S}$ for both normal force and shear force happens when training the force prediction model. The corrected force label $F^{SC} = \{F_{normal}^{SC}, F_{shear}^{SC}\}$ can be obtained when loading the force-image pair data. Specifically, we use contact depth d_z to index force f_z^S and f_z^T from the curves of normal force - depth in both the source sensor *S* and the target sensor *T*. Note that, the indentation depth is normalized for its easy to comparing sensors with different indentation depth due to size and material differences. The compensation ratio *r* can then be calculated in an incremental form:

$$r = \frac{f_z^T}{f_z^S} - 1 \tag{16}$$

We introduce two additional hyperparameters: starting depth d_0 ($0 < d_0 < d_{max}$) and correction weight λ ($0 < \lambda < 1$) to control the amount of compensation. The compensation starts when the contact depth d_z exceeds d_0. λ controls the compensation weight. These parameters used in our paper are obtained via grid search (see Supplementary Table 2 and Supplementary Table 3). Thus, the corrected force label F^{SC} can be derived as:

$$F^{SC} = F^S \cdot (1 + \lambda r) \tag{17}$$

Where *r* is r_L in loading phase while r_U in the unloading phasing indexed with the contact location d_z to catch the hytersis property of elastomer:

$$r = \begin{cases} r_L, & \text{if } d_z > d_0 \text{ and } d_z \in L \\ 0, & \text{if } d_z \le d_0 \\ r_U, & \text{if } d_z > d_0 \text{ and } d_z \in U \end{cases} \tag{18}$$

9.4 Choice of Material Priors

We introduce the choice of the two material priors, including elastic modulus E and friction weighting factor γ.

To get the E if not knowing the material property, we can choose below ways to acquire depending on the experimental conditions: (1) traditional materials characterization method with high accuracy but need to dismantle the sensor or break the elastomer; (2) in-situ calibration using force gauges and indenters without dismantling the sensor and breaking the elastomer[14]; (3) in-situ mechanical calibration method without use of force/torque sensors and the need for a robotic arm with a low-cost calibration devices[15]. In our test, the elastomer has non-linear mechanical property, we estimate E by measuring the relationship between applied normal forces F_N and normalized indentation depth d_z using a rigid indenter (*prism*, contact area 5×7 mm) referring to equation (6). We vertically move the indenter from a non-contact position to place near the maximum depths of sensors: GelSight (1mm), uSkin (1mm), TacTip (4.5mm). We measure the force response both in loading and unloading stage in different indentation depth due to hysteresis property. Then, we normalize indentation depths to the range of 0 to 1 so that those images collected in a step of 0.2 mm (25% of maximum depth 1mm) in GelSight and uSkin can be paired with TacTip with a step of 1.125mm (25% of maximum depth of 4.5mm). The curves are then fitted with a two-degree polynomial using the mean and variance values during three indentations shown in Supplementary Fig. 9C and 15A.

However, friction weighting factor γ is hard to calibrate which involves many variables. Here, we provide empirical factors for γ only using in the real-time deployment stage to compensate the differences in geometry g, tangential shift u, modulus ratio E^* / G^* (effective Poisson ratio) and the coefficient of friction μ. In the training stage, we only compensate for the shear force with E and set γ as 1, which is verified good performance in improvement of shear force in our experimental results.

To further understand the complexity of compensating shear force and the choice of our empirical factors, we exam the statistical relationship between shear force F_S and shear displacement δ (0-1mm) from both homogeneous sensors (see Supplementary Fig. 9B) and heterogeneous sensors (see Supplementary Fig. 15B). We use the “sphere_s” indenter (Supplementary Fig. 1A, diameter of 8mm) to apply forces in different depth and direction by referring to our data collection trajectory. This resulted curve (Supplementary Fig. 9B) matches the relationship shown in [16] with shear and slip stages. This curve can be further processed to the relationship between F_S / F_N and shear displacement δ (Fig. 5C), which implies the coefficient of friction μ between the contact pair of indenter and elastomer. From the curves of F_S - δ (Supplementary Fig. 9B) in homogeneous sensors, we can see that even in the same shear displacement, the F_S varies due to difference in hardness although we use same materials for the pigment

layer on the surface. That explains the necessity of compensate $\frac{E^T}{E^S}$ in the shear force. On the other hand, the mean coefficient of friction μ varies from 0.4-0.7 (Supplementary Fig. 16B) if we only look at the slip region[16] (displacement of 0.6-0.8mm) from curves of F_S / F_N - δ (Fig. 5C). The variation range expands from 0.3 to 0.8 (minimum and maximum values in the slip region of Fig. 5C) when considering the standard variation. Similar mean coefficient of friction μ (0.5-0.7) and variation range (0.3-0.9) can be found in heterogeneous sensors (see mean values in Supplementary Fig. 16A and variation range in Supplementary Fig. 15B). This variation further validates that the shear force is further affected by differences in geometry, hardness et.al. Hence, to compensate γ, we empirically choose friction weighting factor γ as 2 for homogeneous translation while 3 for the heterogeneous translation by referring to the variation range of coefficient of friction.

Text 10. Performance on sensor arrays with pressure sensing

For sensor arrays, whether resistive, capacitive, or magnetic, we can map the tactile response in normal direction to marker size (see Supplementary Fig. 11), while response in shear into marker displacement if available, such as uSkin. If only z-axis signals are available, we represent the normal force by only using marker size. The reference marker image used for mapping tactile response from sensor array can be designed in varied marker size, density and distribution. The mapping sensitivity of the marker changes in size and displacement based on the reference marker image can also be adjusted by parameters when converting from the electrical changes (see Supplementary Fig. 12A).

To test the transferability of our model among sensor arrays with only pressure sensing, we downsample the $4 \times 4 \times 3$ multichannel signal of uSkin (three-axis) into 4×4 channels only with measurement from the z-channel in each texel. Then, in the signal-to-marker stage, the predefined markers can only change the size due to external forces without the displacement in x and y axis. Based on this, we train our model using the modality, named uSkin (z-axis), to transfer across flat-surface vision-based tactile sensor (GelSight, marker pattern D-I) and curved-surface vision-based tactile sensor (TacTip, palm shape).

As shown in Supplementary Fig. 25, the model successfully transfers the images from the GelSight (D-I) and TacTip (palm) to uSkin (z-axis). In the contact area, only marker size changes are observed in the generated images similar to the target images. We then use the transferred uSkin (z-axis) image to train the force prediction model and deploy in real-time to compared it with ATI nano17 F/T sensor by using three daily objects (see Supplementary Fig. 26 and Supplementary Video 3). Due to the loss of

information in x and y directions, it is known that the sensor array with only z-axis sensing capabilities is hard to get precise shear force prediction. In this case, we only show its capability to predict normal force in real time by using the transferred model from a vision-based tactile sensor. We can see that the sensor is still able to respond to high-accuracy forces in real-time. This experiment verifies our model is also generalizable to modality without shear force and intrinsically no marker shear displacement.

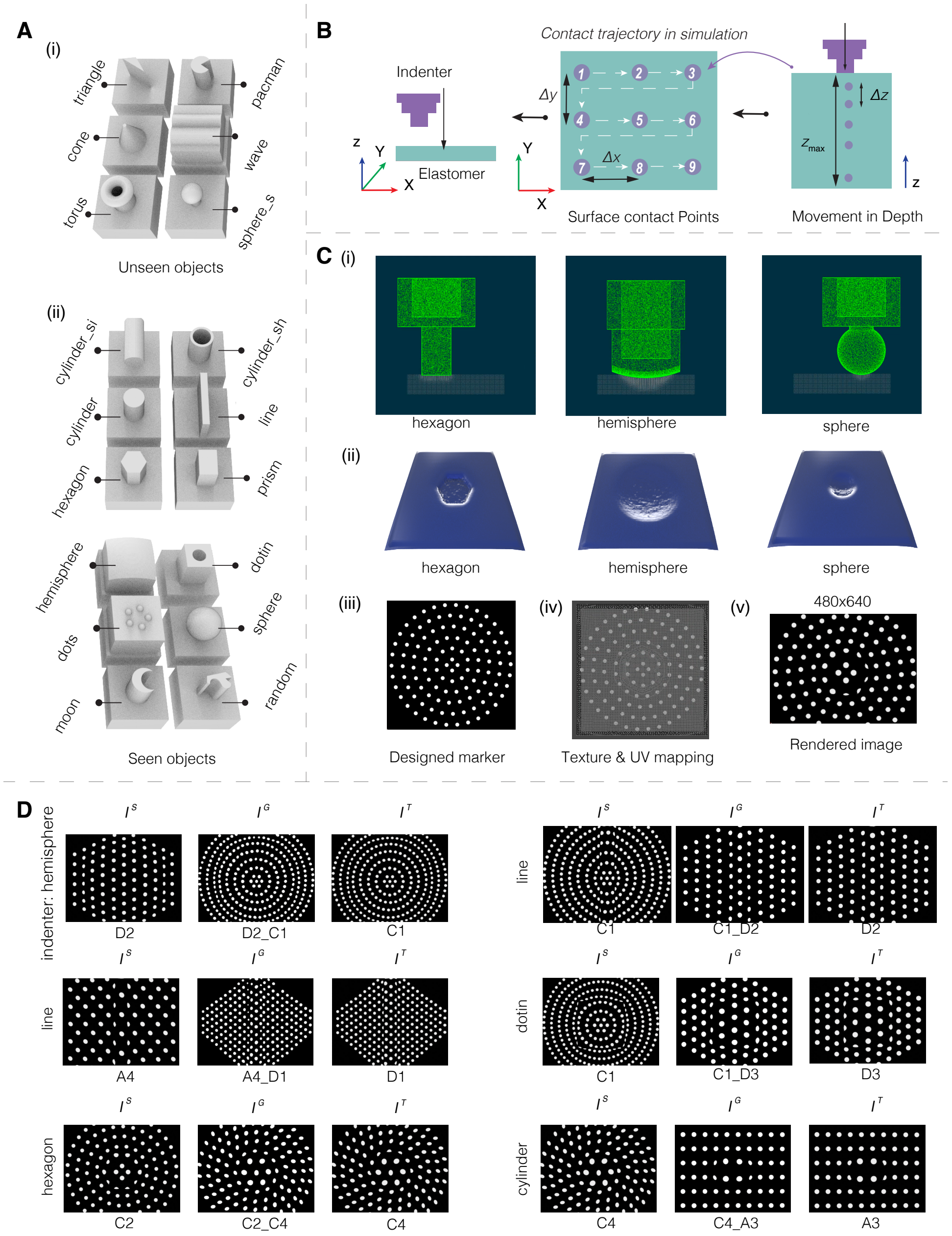

Supplementary Figure 1. Simulation framework for marker deformation. (A) Primitive indenters are categorized into unseen (i) and seen (ii) objects. **(B)** Data collection trajectories are used in simulation. **(C)** Marker deformation (i) is simulated in Tacchi, followed by the generation of a deformed mesh (ii), application of designed marker patterns (iii), rendering of deformed images in Blender (iv), and visualization of the final deformed images (v). **(D)** Examples of six randomly selected marker-to-marker translation results using simulated data.

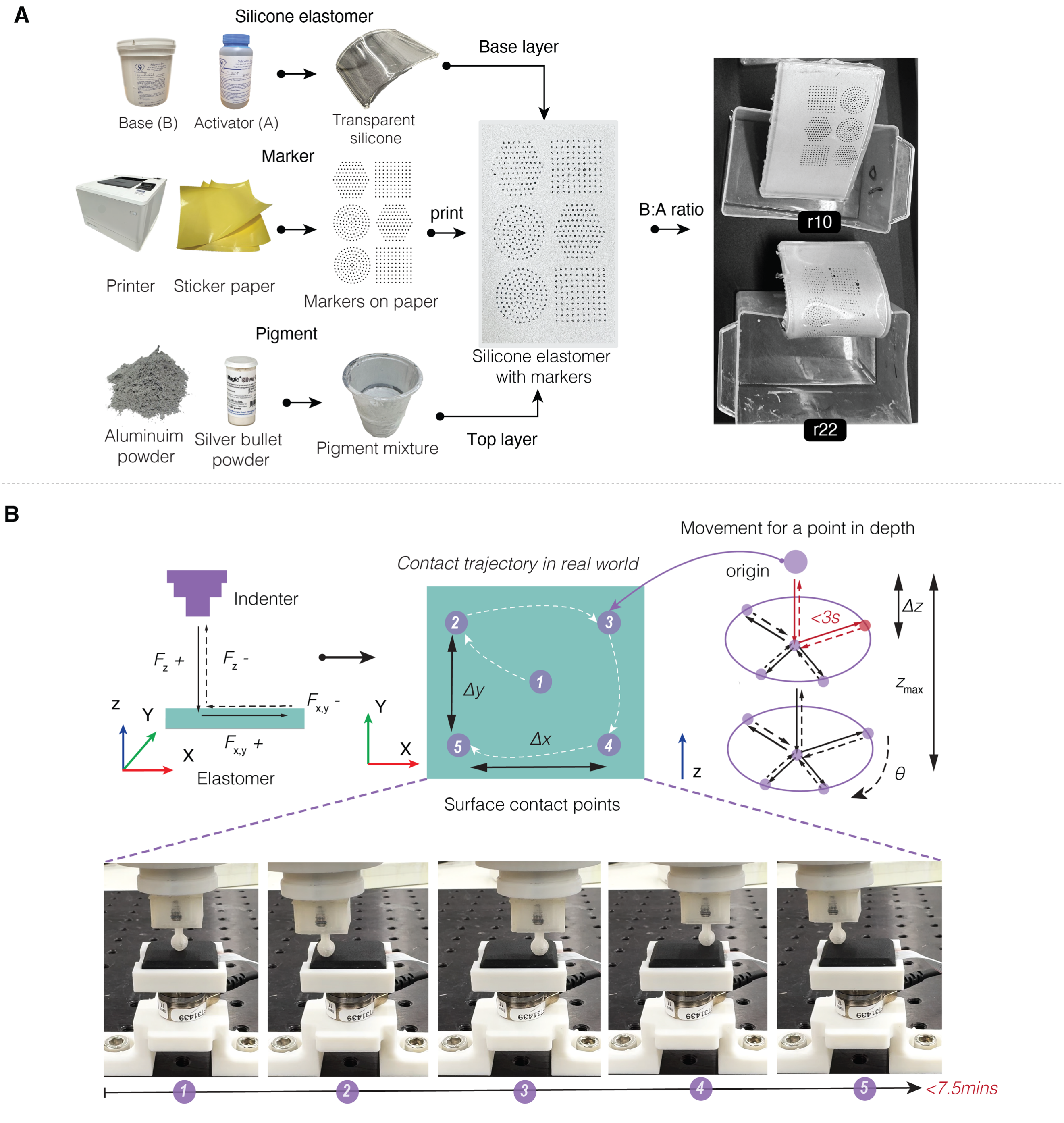

Supplementary Figure 2. Data collection in real world. (A) Fabrication process of soft skin with varied markers and hardness. **(B)** Data collection trajectories are used in real world. An elastomer is divided into five surface contact points. For each surface point, the indenter will move in depth with four actions: moving downward, moving lateral outward, moving lateral inward, and moving upward for a contact point. The movement for a point in depth takes less than 3s, while the total time cost is less than 7.5 mins.

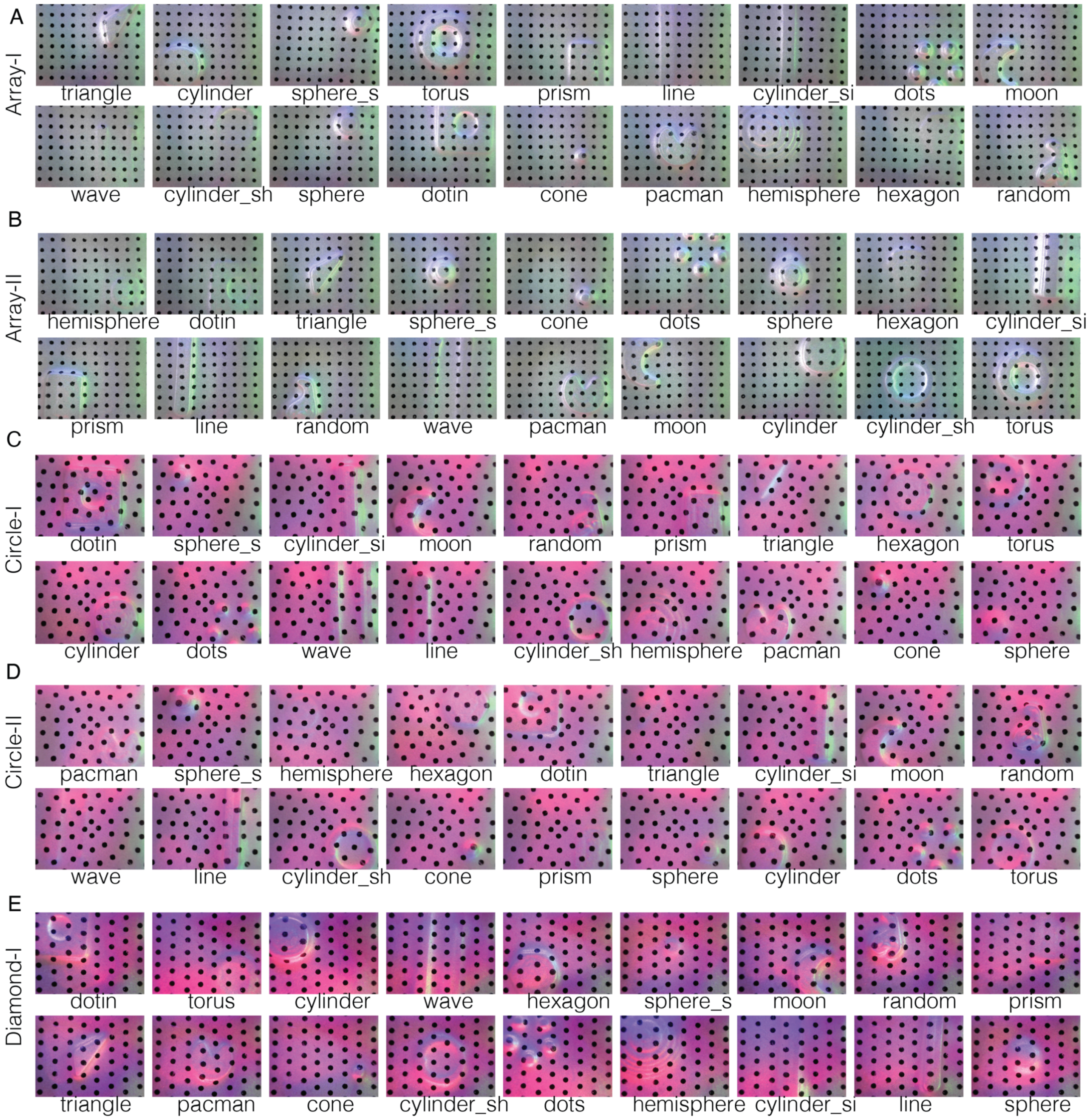

Supplementary Figure 3. Examples of randomly selected RGB images collected from five homogeneous GelSight sensors (**A-E**) deformed by eighteen primitive indenters in different depth and position.

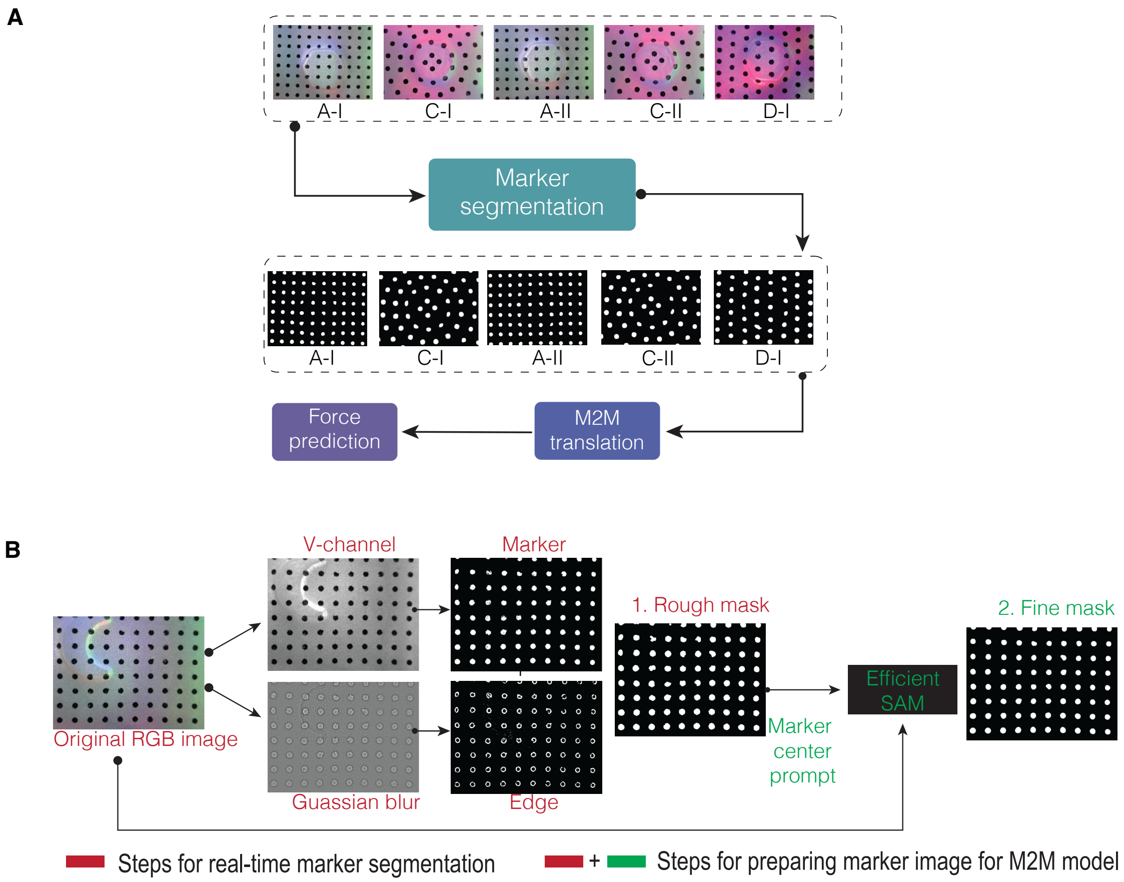

Supplementary Figure 4. Marker-to-marker translation results for homogeneous sensors. (A) Pipeline for homogeneous translation. **(B)** Marker segmentation pipeline for the GelSight sensors, including rough processing and fine processing. The rough process can run in real-time more than 29.6Hz together with force prediction when the FPS of the camera is 30 Hz.

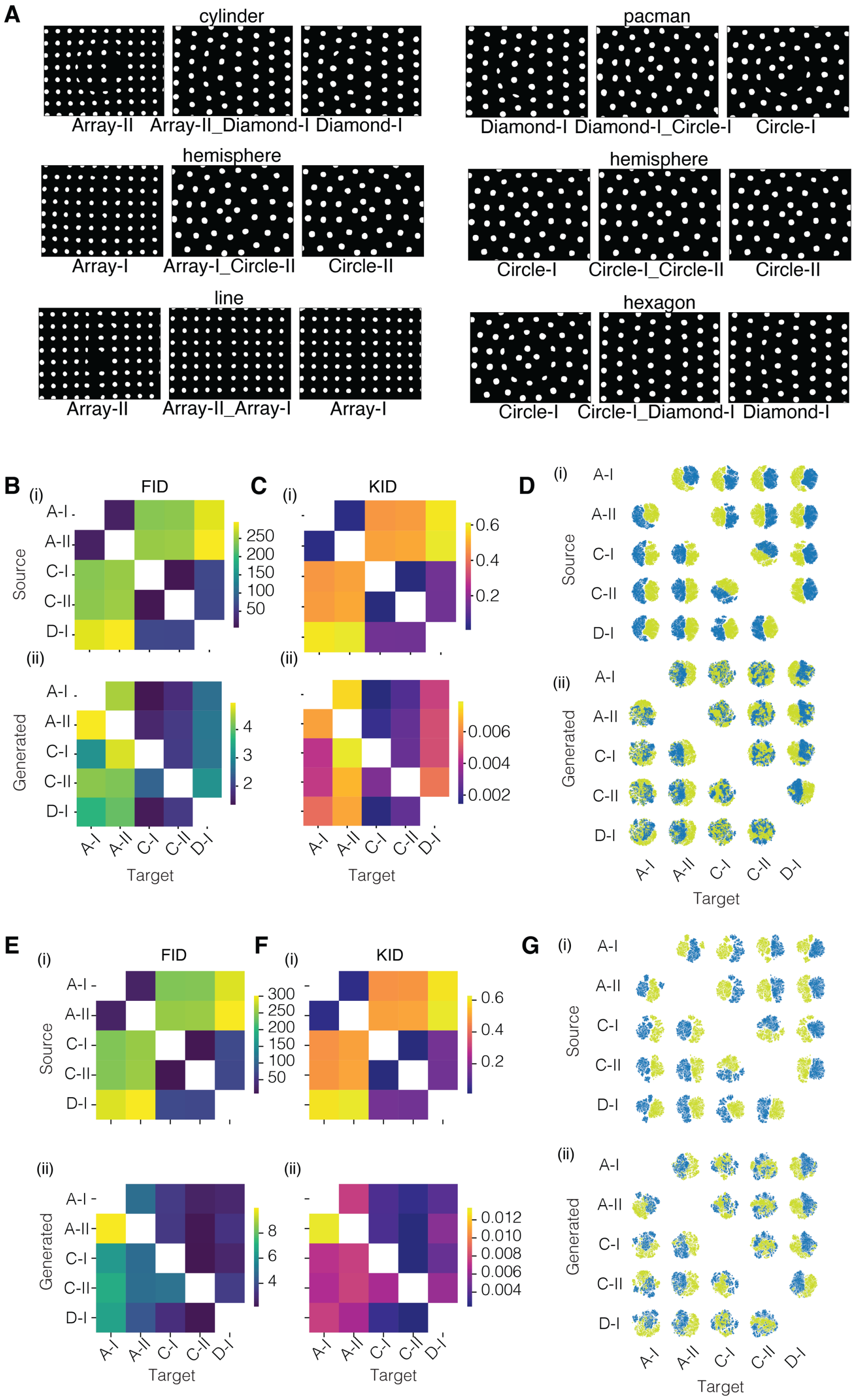

Supplementary Figure 5. Marker-to-marker translation results for homogeneous sensors. (A) Randomly selected marker images after marker-to-marker translations across five GelSight sensors. Source image, generated images and target images are in the first to third column respectively. Applied indenters are labelled in the tip. **(B-C)** Heatmaps of the FID (B) and KID (C) results before (i) and after (ii) using M2M model in seen group. **(D)** Feature space visualization before and after applying M2M model, showing improved alignment in feature spaces, tested with objects in seen group. **(E-F)** FID (E) and KID (F) before (i) and after (ii) using M2M model, tested with objects in unseen group. **(G)** Visualization of the feature space using t-SNE before (i) and after (ii) applying M2M model, tested with objects in unseen group. The row labels are source sensors, while the columns are target sensors.

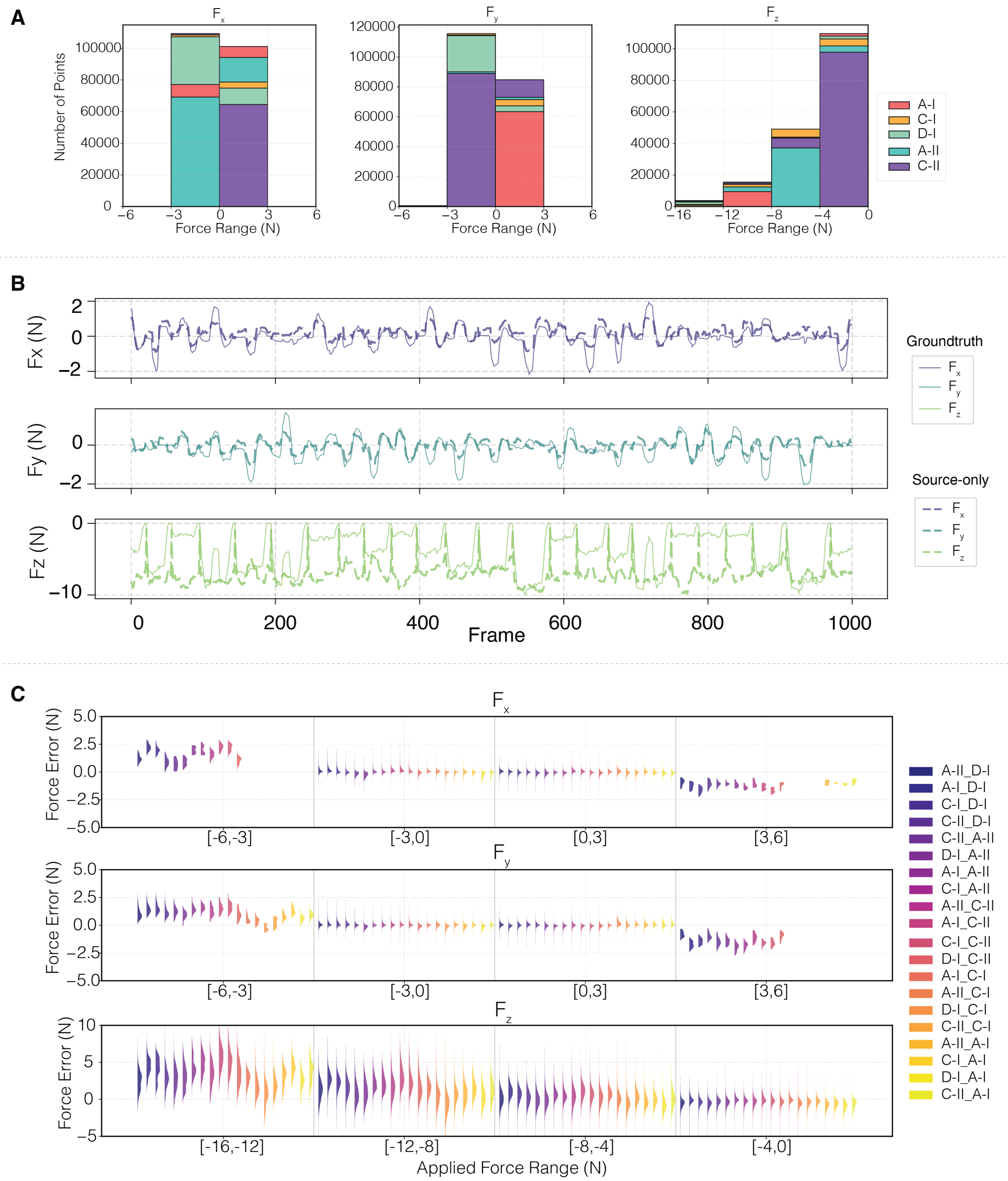

Supplementary Figure 6. Force prediction results for homogeneous sensor translation. (A) Distribution of data points collected across different force ranges for normal and shear forces across five sensors. **(B)** Real-time force prediction over 1000 frames when using source-only method in group *A-II_D-I*. **(C)** Half-violin plots showing force prediction errors for twenty sensor combinations across varying force ranges.

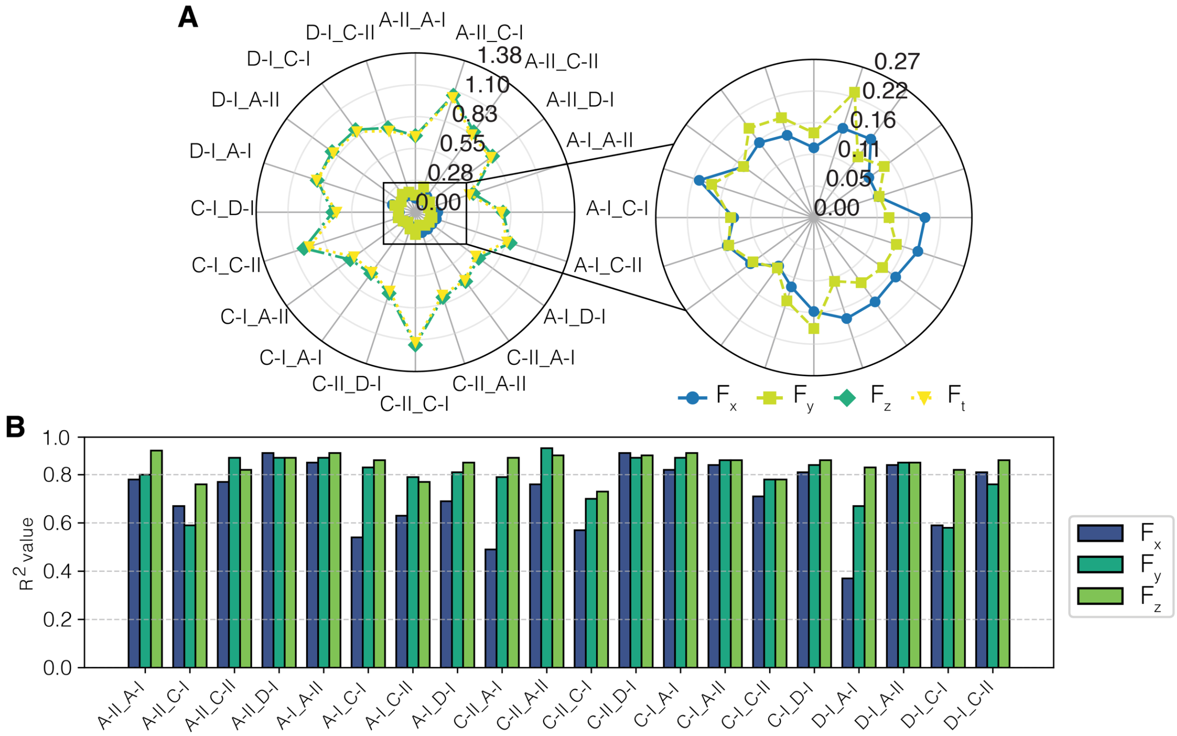

Supplementary Figure 7. Force prediction results for homogeneous sensor translation in unseen group. (A) Radar plots of MAE in homogeneous translation tested with objects in unseen group. **(B)** Histogram of R^2 values for homogeneous translation results across five sensors tested with objects in unseen group.

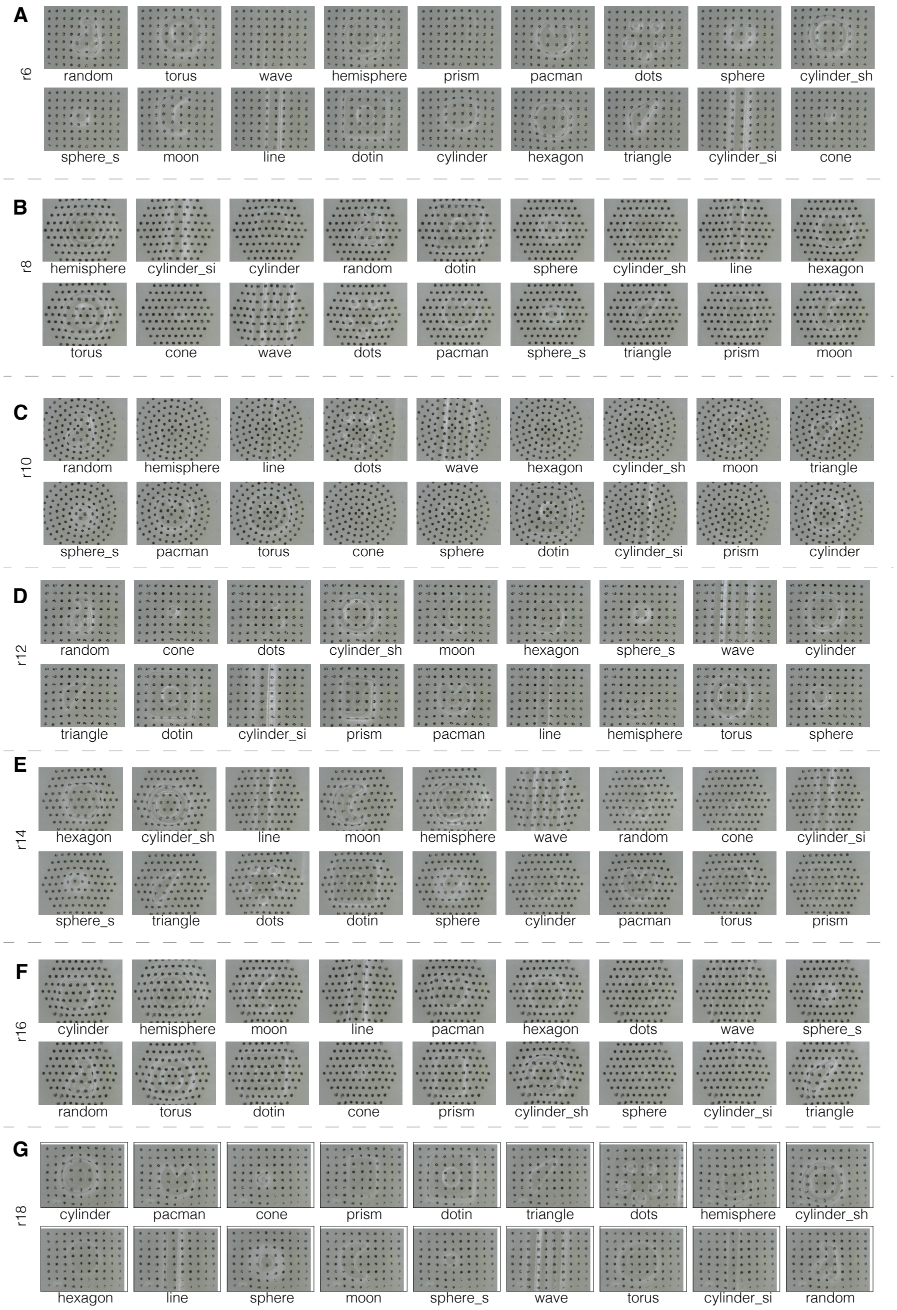

Supplementary Figure 8. **(A-G)** Examples of collected tactile images randomly selected from seven GelSight sensors with varying skin hardness, deformed by eighteen indenters. Note that, some images may not show clear geometry of the indenters as those are with small indentation depth.

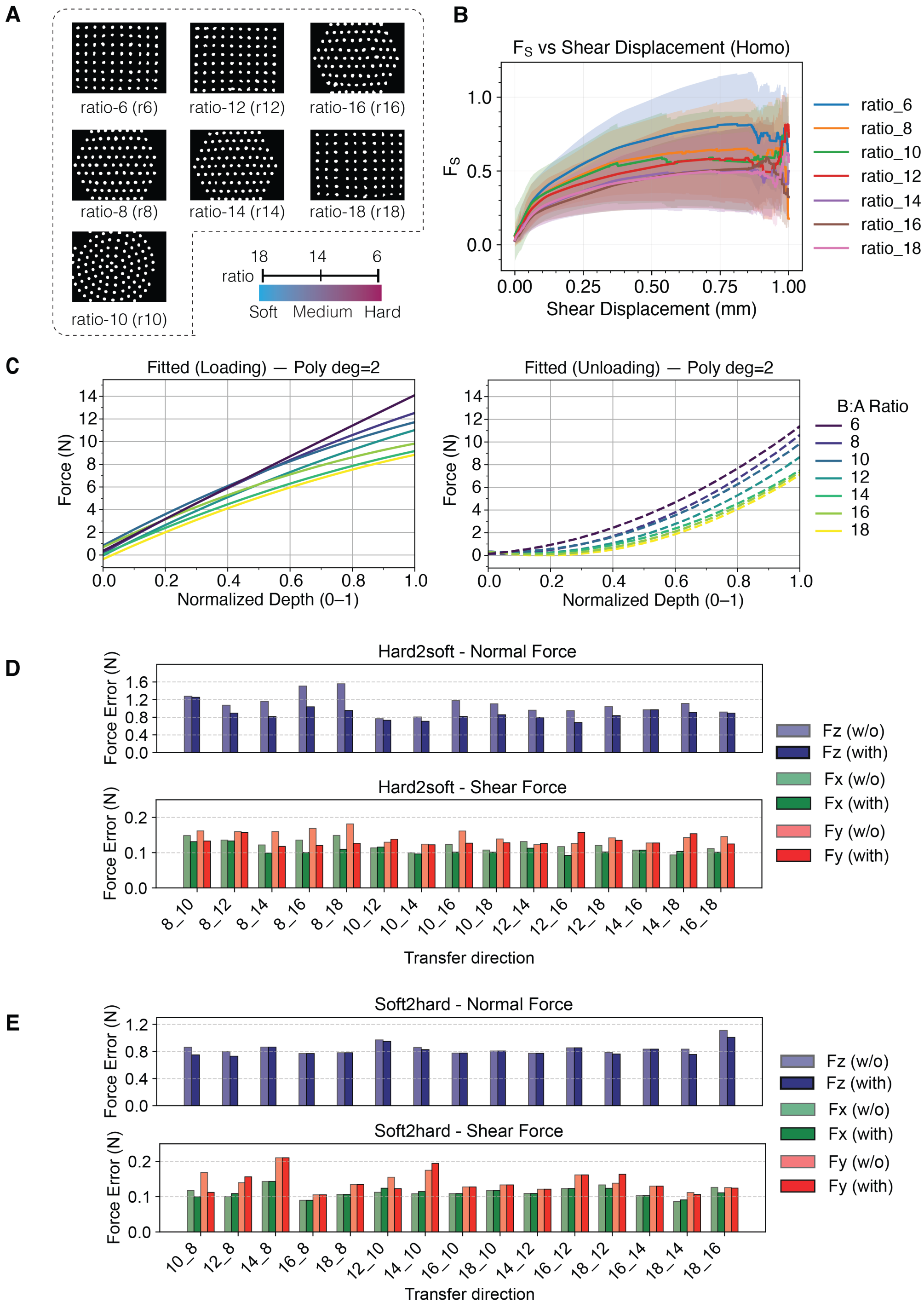

Supplementary Figure 9. Material compensation for force prediction. (A) Marker-based elastomers fabricated with seven base-to-activator ratios, where higher ratios yield softer elastomer and lower ratios produce harder elastomer. **(B)** Relationship of shear force and shear displacement for seven elastomers (± 1 SD). **(C)** Relationship of force and normalized depth for seven elastomers with two-degree polynomial fitted lines in loading and unloading stage. **(D-E)** Force prediction errors for GenForce models without (w/o) and with material compensation in hard-to-soft group (D) and soft-to-hard group (E).

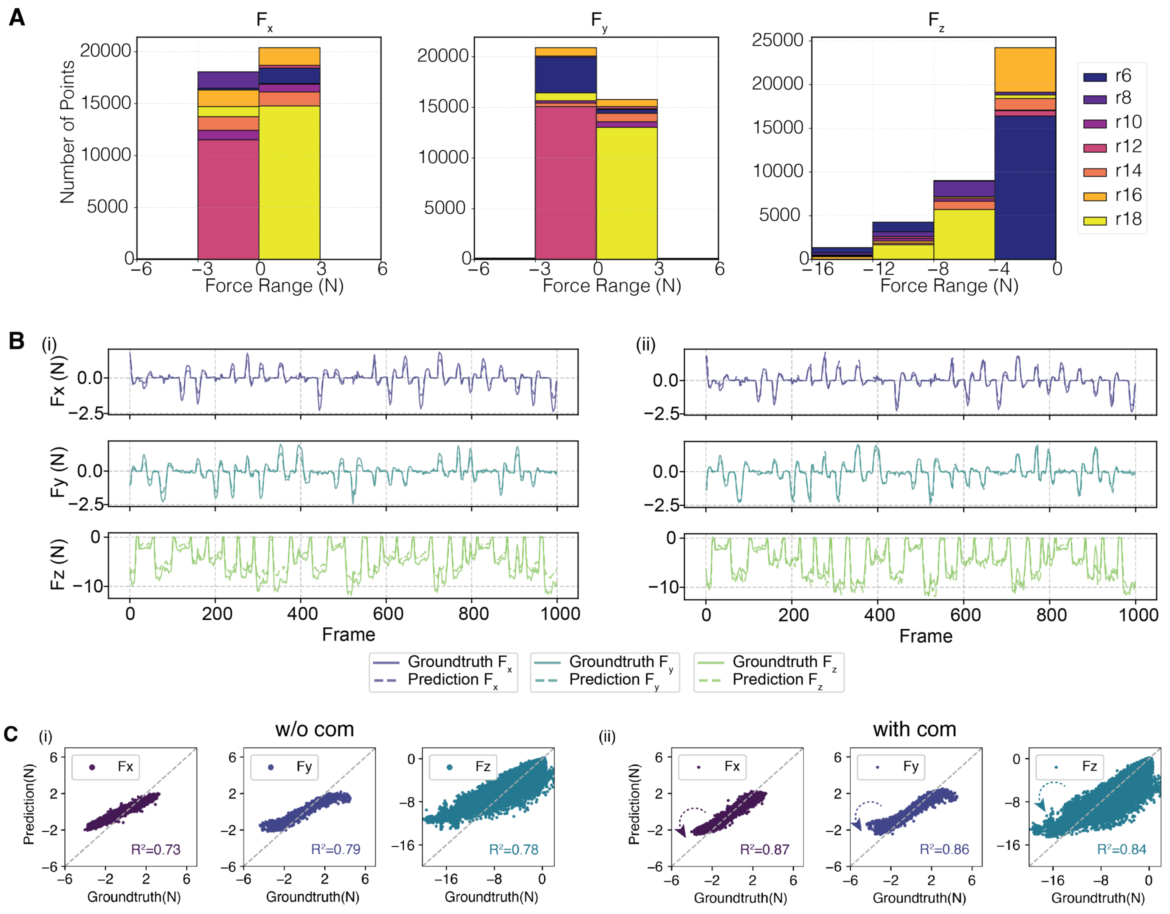

Supplementary Figure 10. Material compensation results for force prediction. (A) Distribution of data points collected across varying force ranges (normal and shear forces) for seven sensors. **(B)** Real-time comparison of force prediction over 1000 frames using the GenForce model without (i) and with (ii) material compensation. The source domain is a GelSight sensor with a silicone elastomer named r16 (soft, diamond-like marker pattern), while the target domain is a GelSight sensor with a silicone elastomer named r6 (hard, array-like marker pattern). Frames are randomly selected from the test dataset. **(C)** Fit of force prediction to ground truth demonstrates the effectiveness of material compensation when transferring from a sensor with r18 (soft) skin to r6 (hard) skin without (i) and with (ii) material compensation.

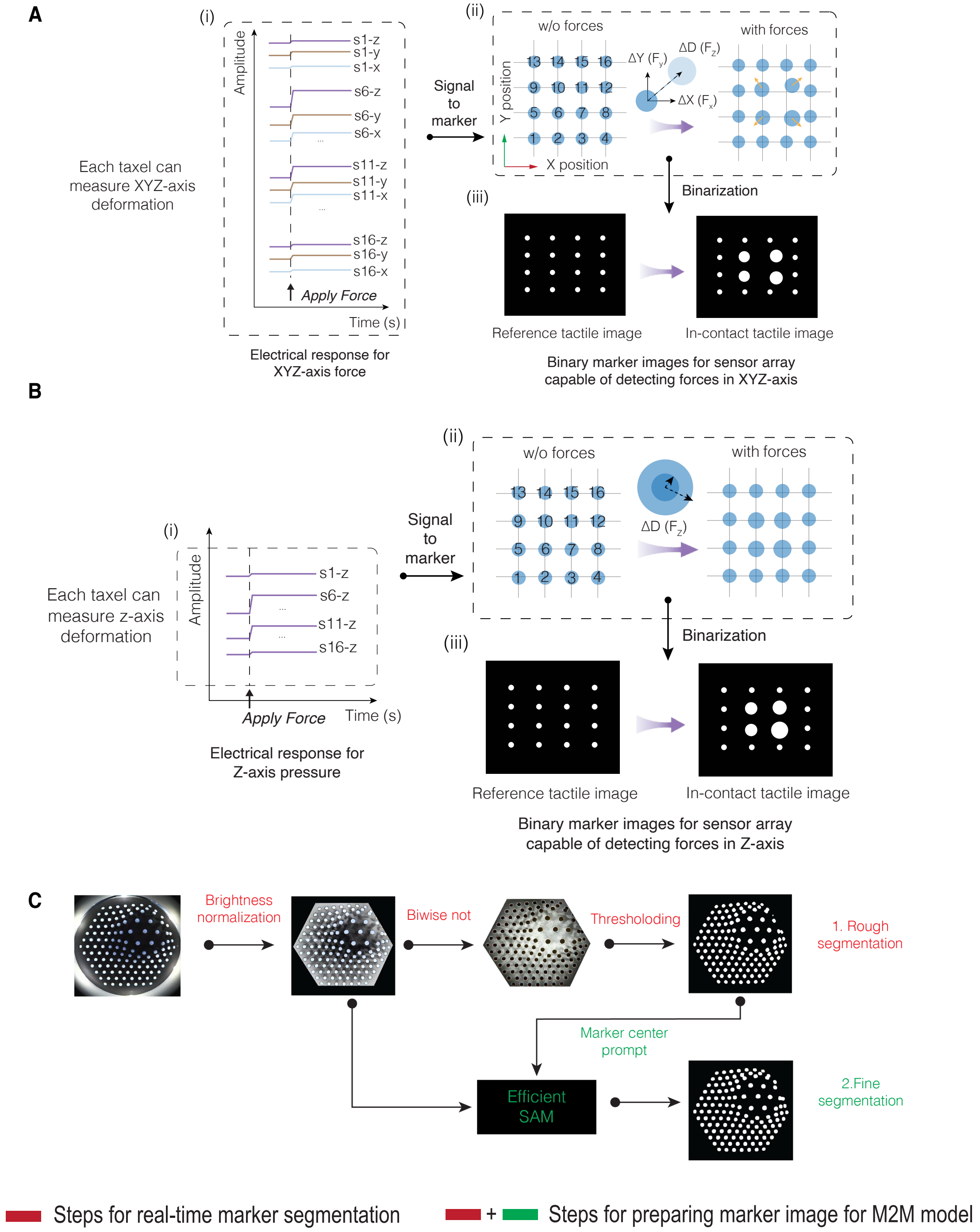

Supplementary Figure 11. Marker conversion for sensor arrays and TacTip. (A) Signal-to-marker process for sensor arrays capable of sensing deformation in three-axis in each taxel. **(B)** Signal-to-marker process for sensor arrays capable of sensing deformation in z-axis in each taxel**. (C)** Marker segmentation pipeline for TacTip. The rough process can run in real-time more than 29.6Hz together with force prediction when the FPS of the camera is 30 Hz.

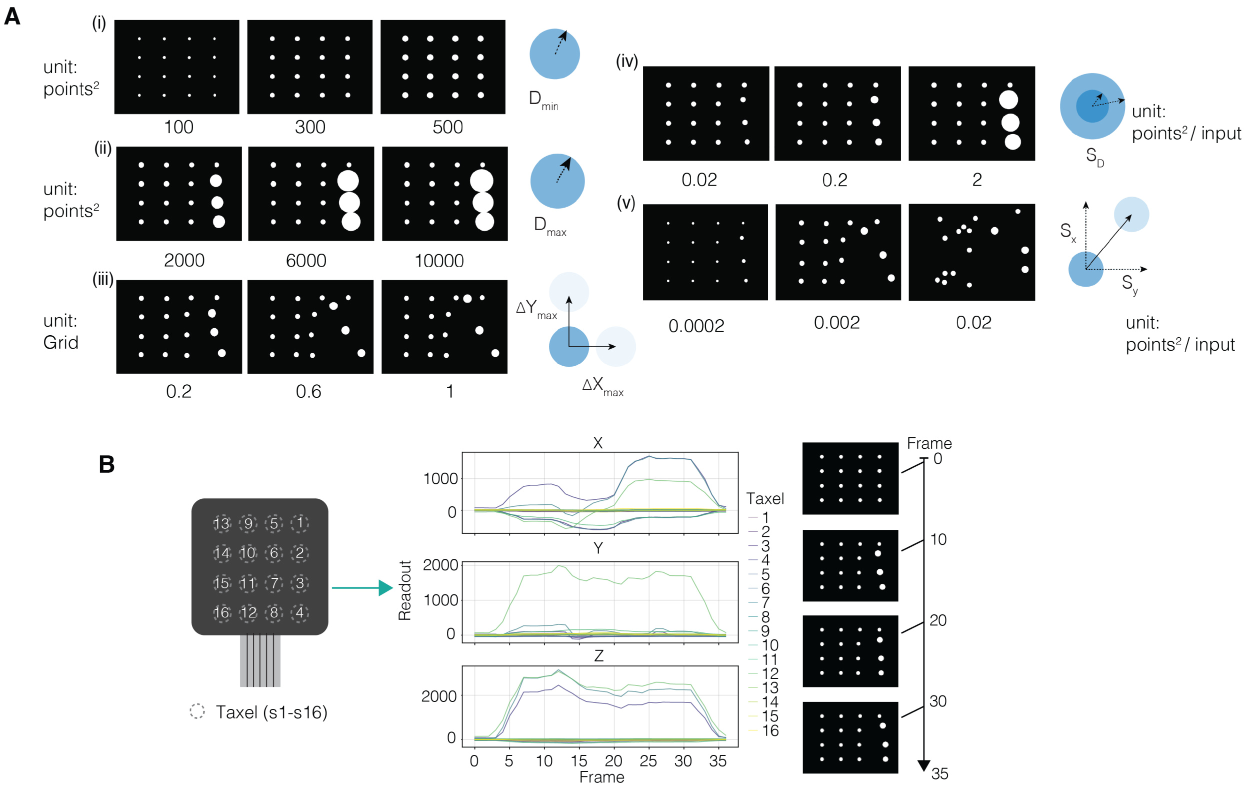

Supplementary Figure 12. Signal-to-marker for uSkin. (A) Seven hyperparameters used for signal-to-marker conversion in sensor arrays capable of sensing three-axis deformation in each taxel. These hyperparameters include marker size $D_{\min}$ (i) and $D_{\max}$ (ii), marker offset $\Delta X_{\max}$ (iii) and $\Delta Y_{\max}$ (iii), sensitivity S_D (iv) for marker size change, and sensitivity S_x, S_y (v) for marker offset changes. **(B)** Illustration of converting multichannel electrical signals from uSkin (three-axis) to binary marker images.

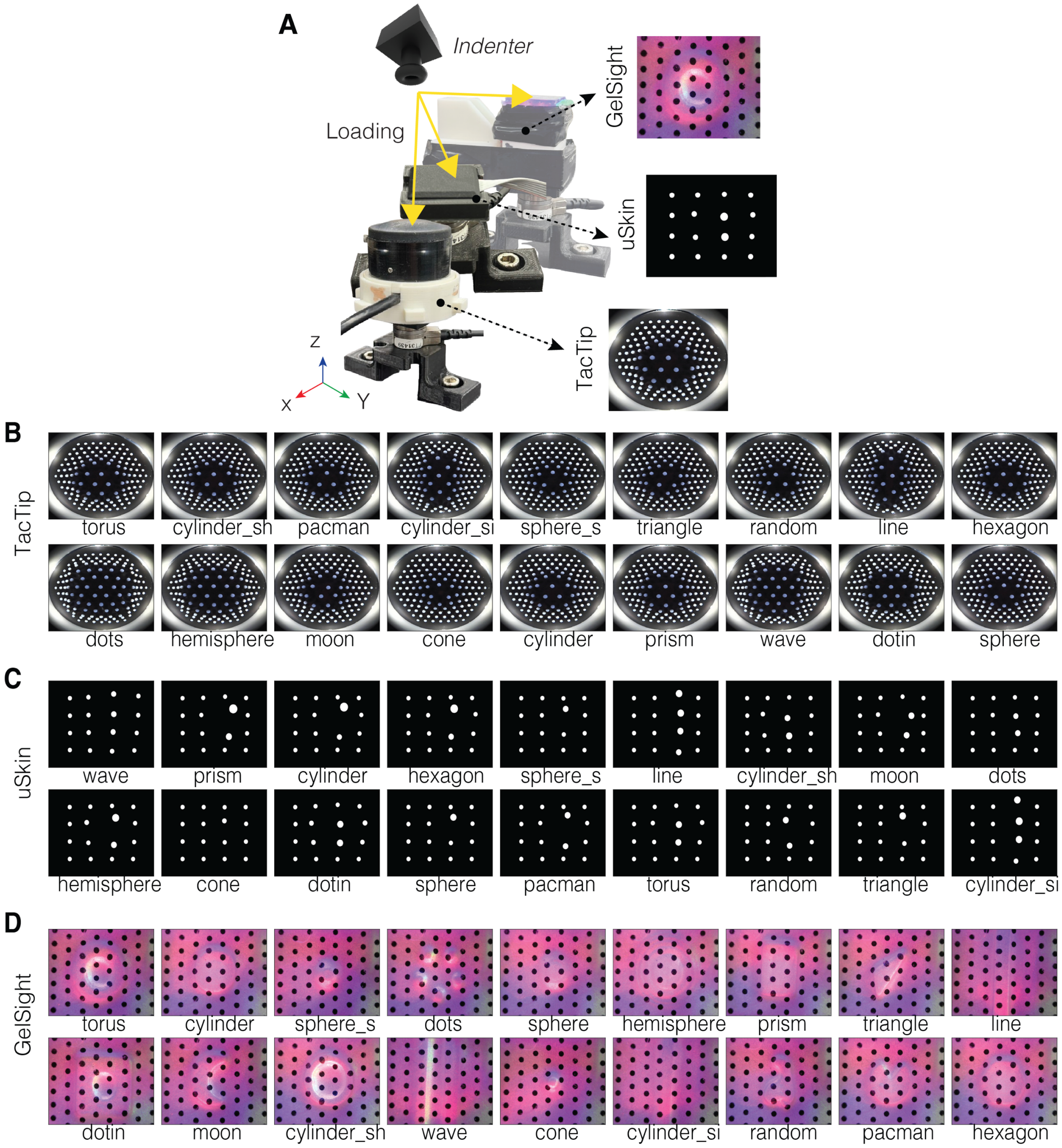

Supplementary Figure 13. Data collection and visualization. (A) Data collection setup for heterogeneous sensors. **(B-D)** Examples of tactile images randomly selected from three heterogeneous tactile sensors: TacTip (i), uSkin (ii), and GelSight (iii), deformed by eighteen indenters. Note that, some images may not show clear geometry of the indenters as those are with small indentation depth.

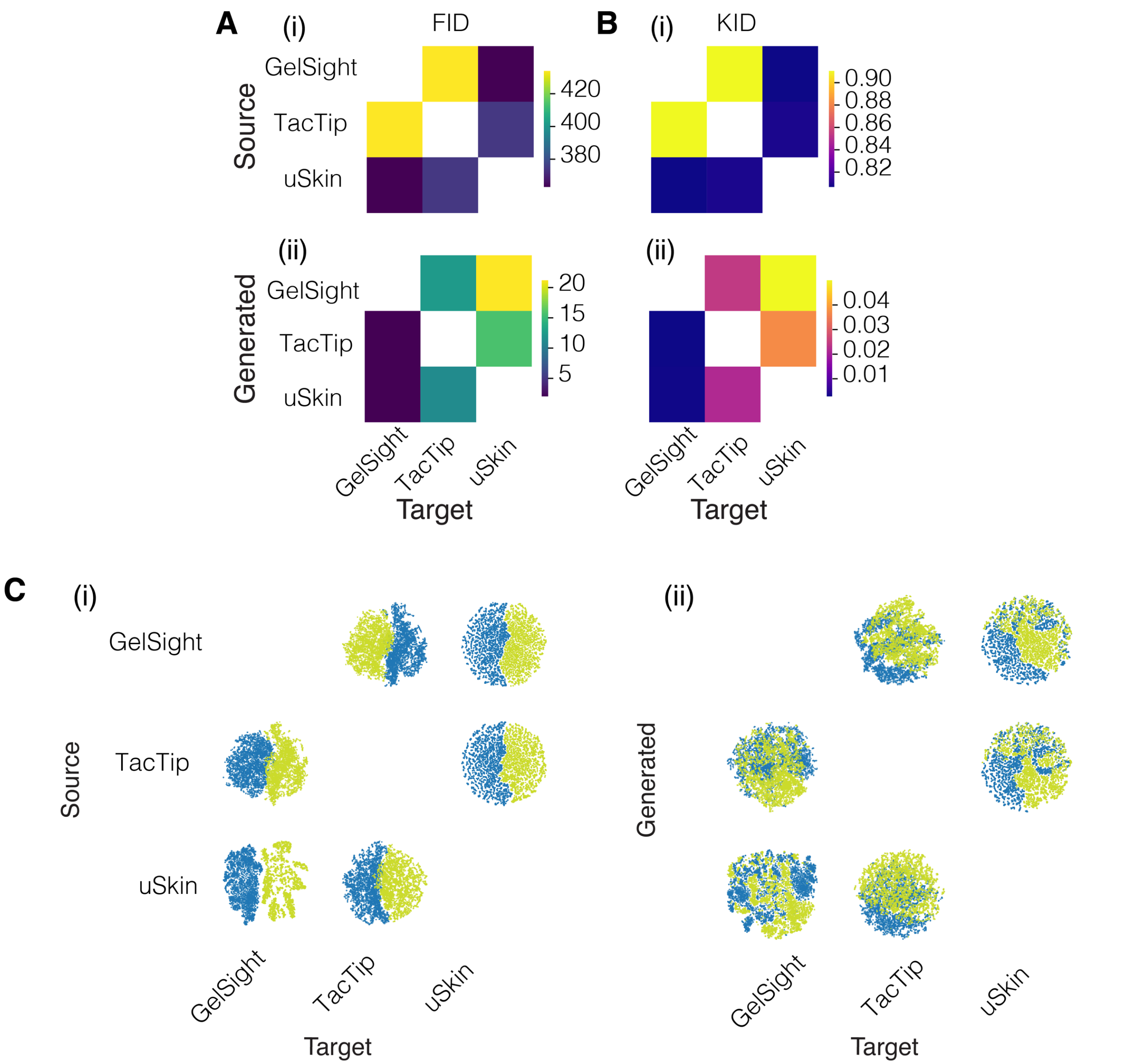

Supplementary Figure 14. Marker-to-marker translation results of heterogeneous sensors. (A-B) FID (A) and KID (B) before (i) and after (ii) using M2M model. **(C)** Visualization of the feature space using t-SNE before (i) and after (ii) applying M2M model.

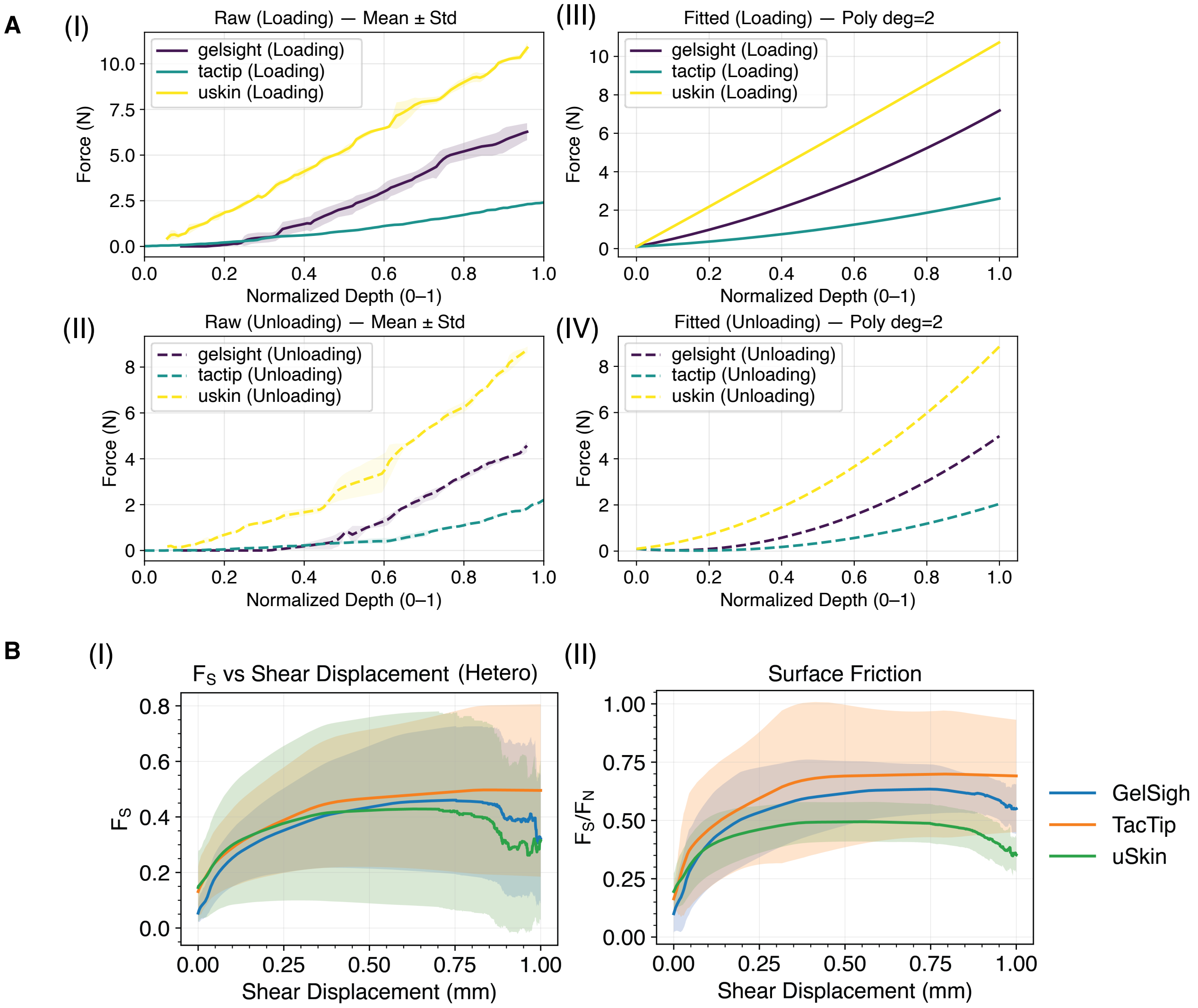

Supplementary Figure 15. Material property of three heterogeneous sensors. (A) Force-normalized depth curve of raw data (i-ii) and fitted data (iii-iv) in loading/unloading stages. Note that fitted curves are fitted with 2-degree polynomials. **(B)** Relationship of shear force and shear displacement (i) for three heterogeneous sensors. The curve (i) is then divvied with normal force to show the relationship of F_S/F_N and shear displacement (ii). All shaded regions are $\pm$1SD.

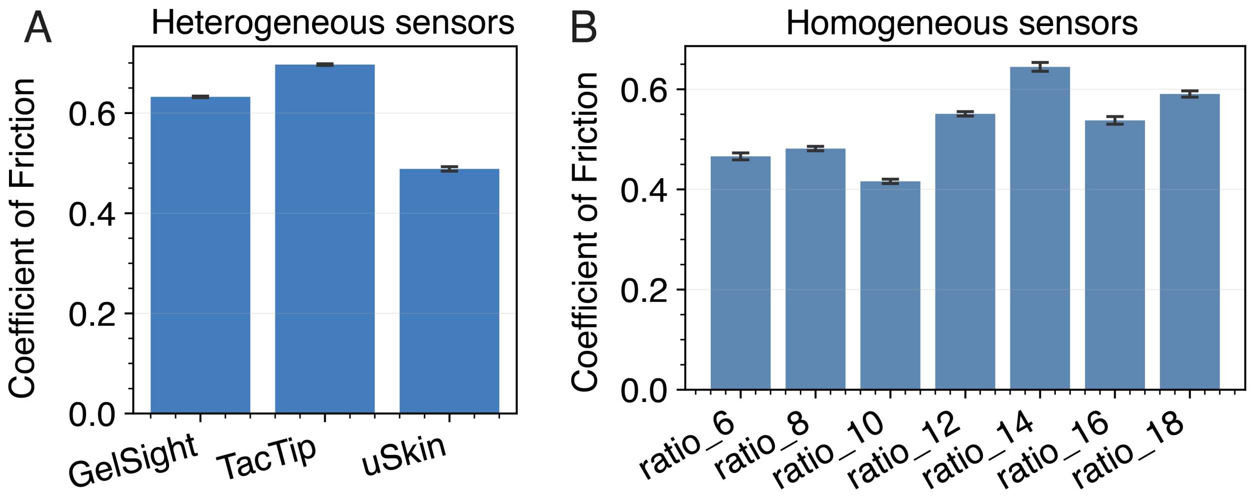

Supplementary Figure 16. Coefficient of friction of heterogeneous sensors (A) and homogeneous sensors (B). The coefficient of friction is calculated by the mean and std from the curve of F_S/F_N to shear displacement in the completely friction area (shear displacement in [0.6,0.8] mm). All error bars are $\pm$1SD.

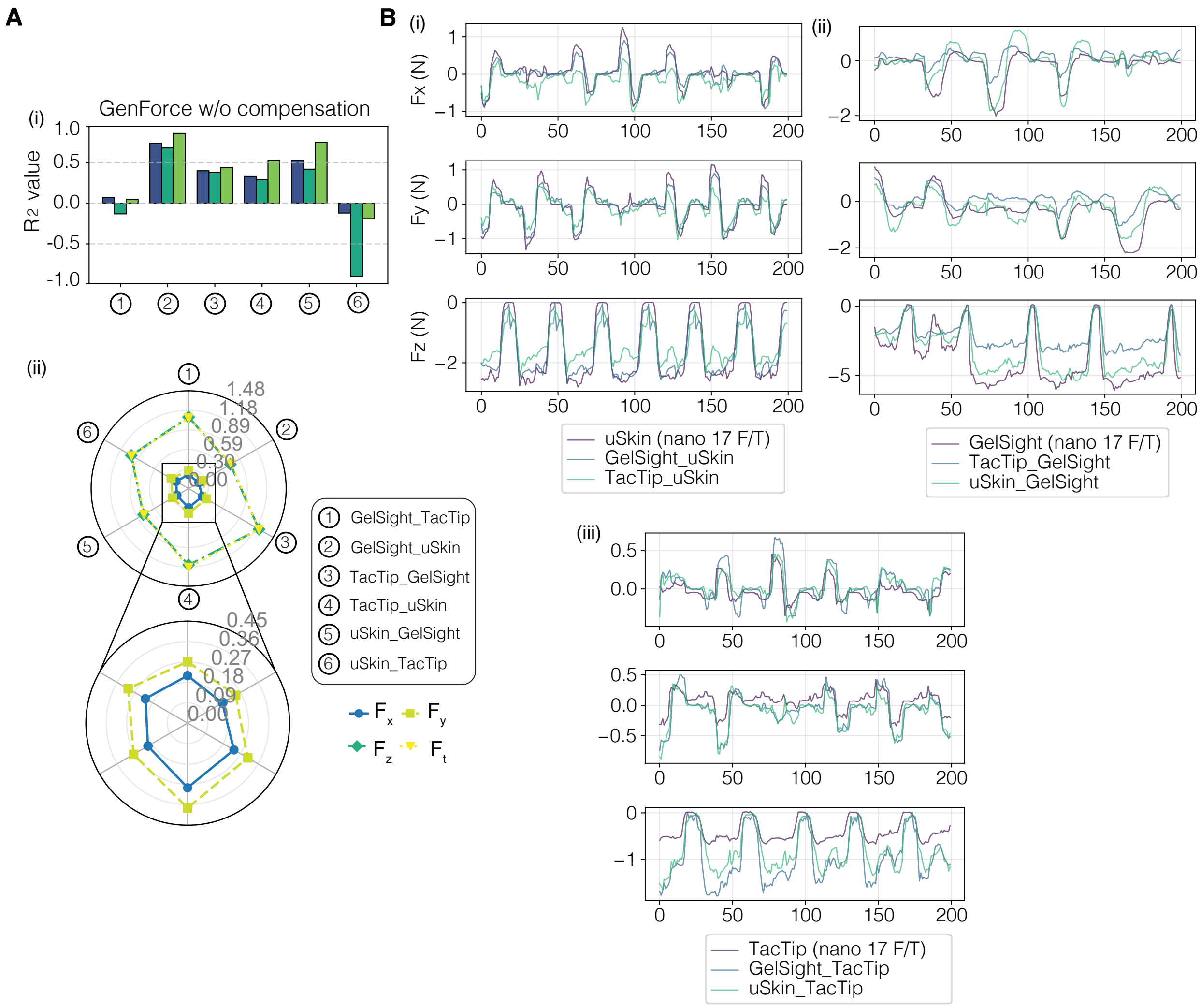

Supplementary Figure 17. Heterogeneous translation results without material compensation. (A) Histogram of R^2 values (i) and radar plot (ii) of MAE after using GenForce without material compensation. **(B)** Visualization of force prediction performance without material compensation when uSkin (i), GelSight (ii), and TacTip (iii) serve as the target domain (from left to right) respectively.

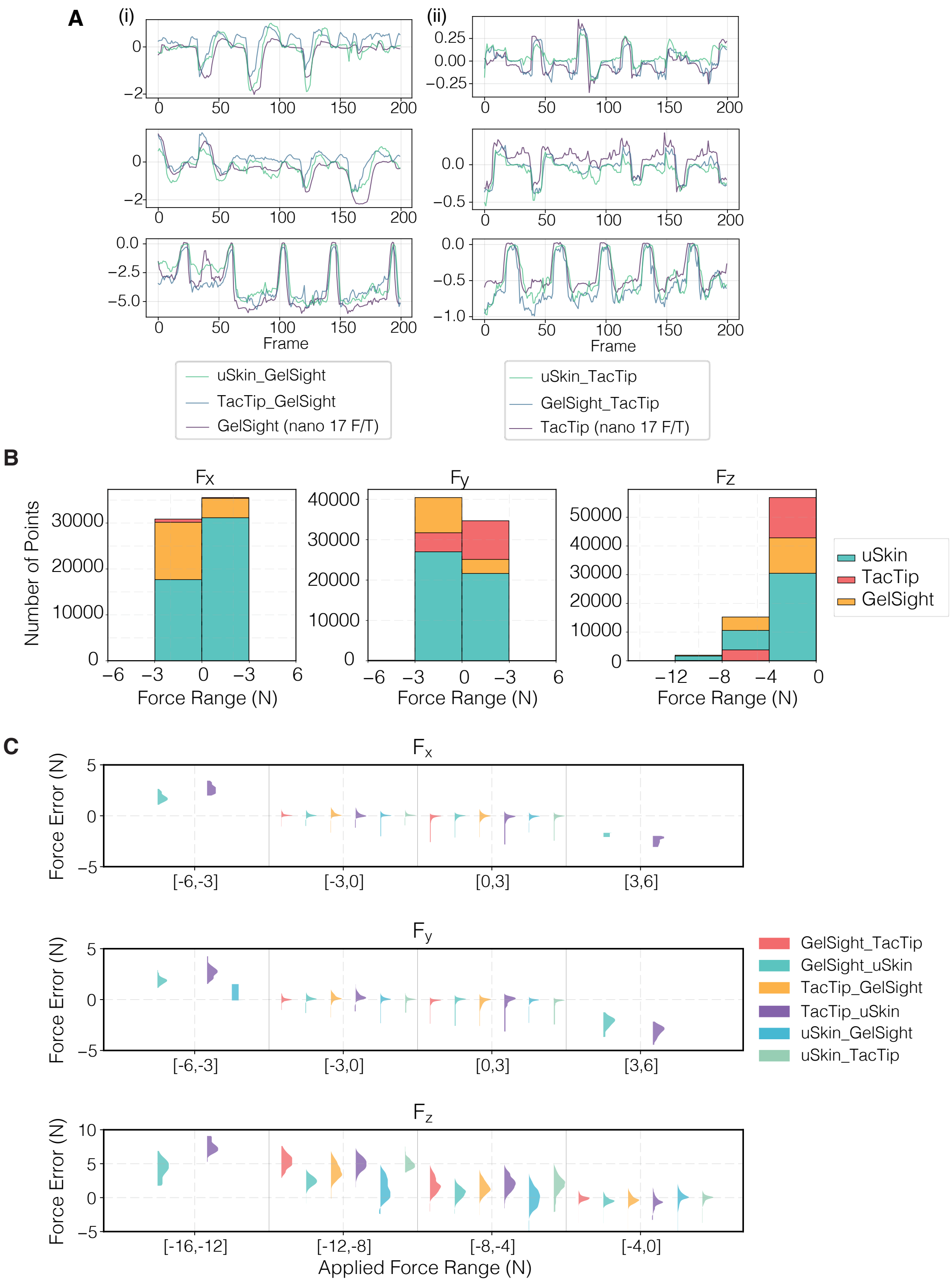

Supplementary Figure 18. Results for heterogeneous translation with material compensation. (A) Real-time demonstration of force prediction when target domains are GelSight (i), and TacTip (ii) after using GenForce model with material compensation. Also see animated version in Supplementary Video 2. **(B)** Distribution of data points collected across different force ranges for normal and shear forces across three heterogeneous sensors. **(C)** Half-violin plots showing force prediction errors for six sensor combinations across varying force ranges after using GenForce and material compensation. It demonstrates larger force error in higher force range. Notably, the performance in the force range of -4 N to 0 N in the normal direction and -3N to 3 N in the shear direction are observed with zero-centered errors.

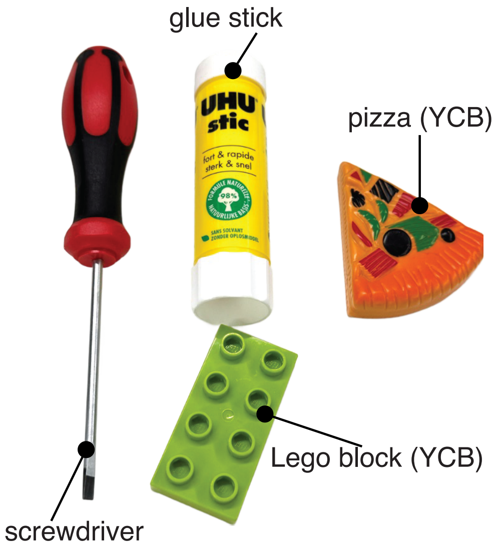

Supplementary Figure 19. Objects used for dynamic force test compared with ATI nano17 F/T sensor in real-time. We use four objects with different materials, sizes and shapes, including a screwdriver, glue stick, Lego block (from YCB dataset) and pizza (YCB).

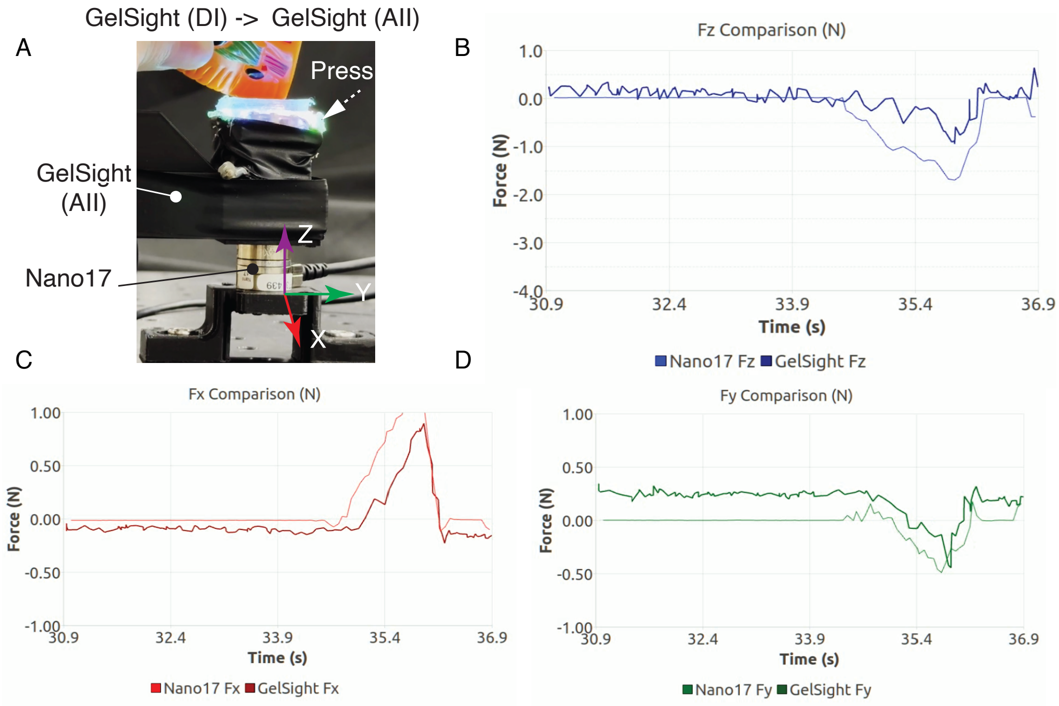

Supplementary Figure 20. Real-time force prediction when pressing on a GelSight sensor. (A) Demonstration of test object, contact event, tactile sensor and nano17. The force model is transferred from a GelSight (D-I) sensor. **(B-D)** Force prediction performance in Z-axis, X-axis and Y-axis respectively.

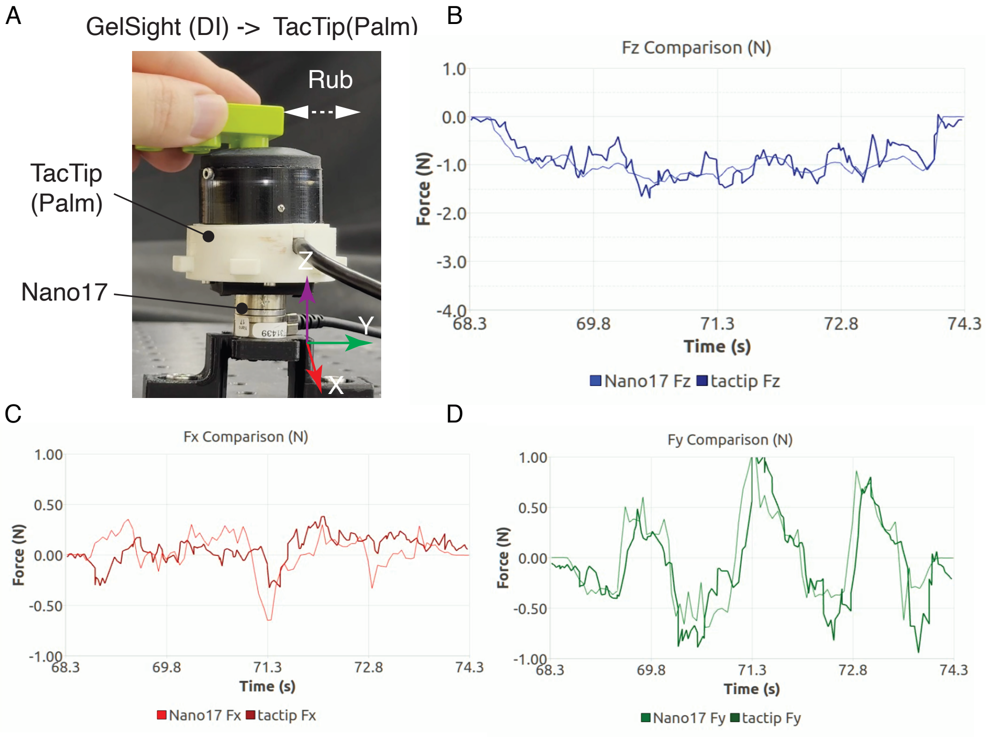

Supplementary Figure 21. Real-time force prediction when rubbing on a TacTip sensor. (A) Demonstration of test object, contact event, tactile sensor and nano17. The force model is transferred from a GelSight (D-I) sensor. **(B-D)** Force prediction performance in Z-axis, X-axis and Y-axis respectively.

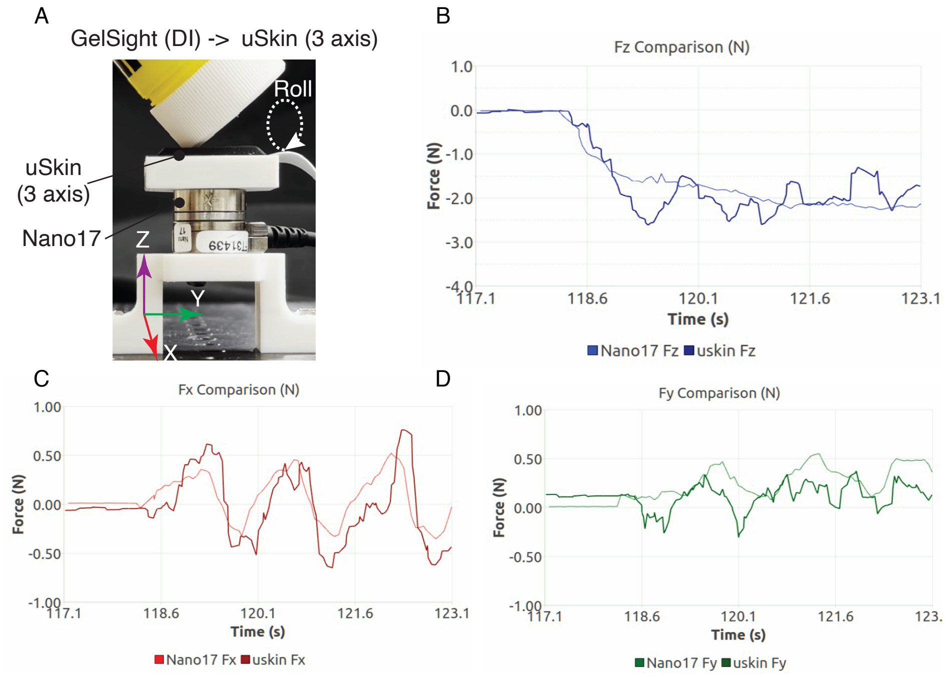

Supplementary Figure 22. Real-time force prediction when rolling on a uSkin (three-axis) sensor. (A) Demonstration of test object, contact event, tactile sensor and nano17. The force model is transferred from a GelSight (D-I) sensor. (B-D) Force prediction performance in Z-axis, X-axis and Y-axis respectively.

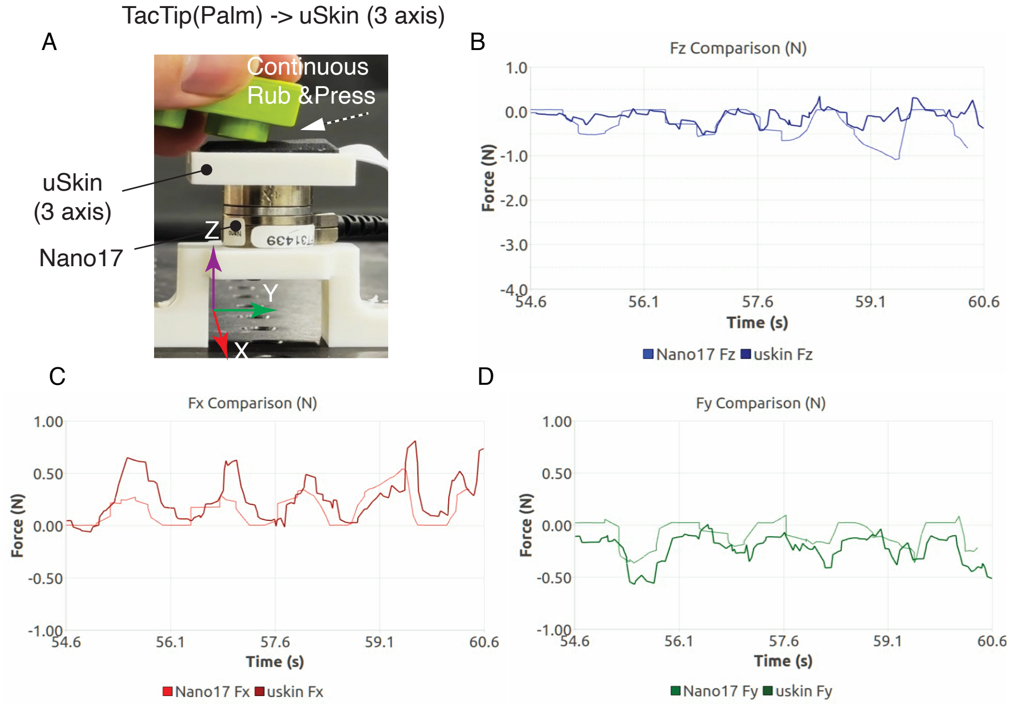

Supplementary Figure 23. Real-time force prediction when continuously rubbing and pressing on a uSkin sensor. (A) Demonstration of test object, contact event, tactile sensor and nano17. The force model is transferred from a TacTip (palm) sensor. **(B-D)** Force prediction performance in Z-axis, X-axis and Y-axis respectively.

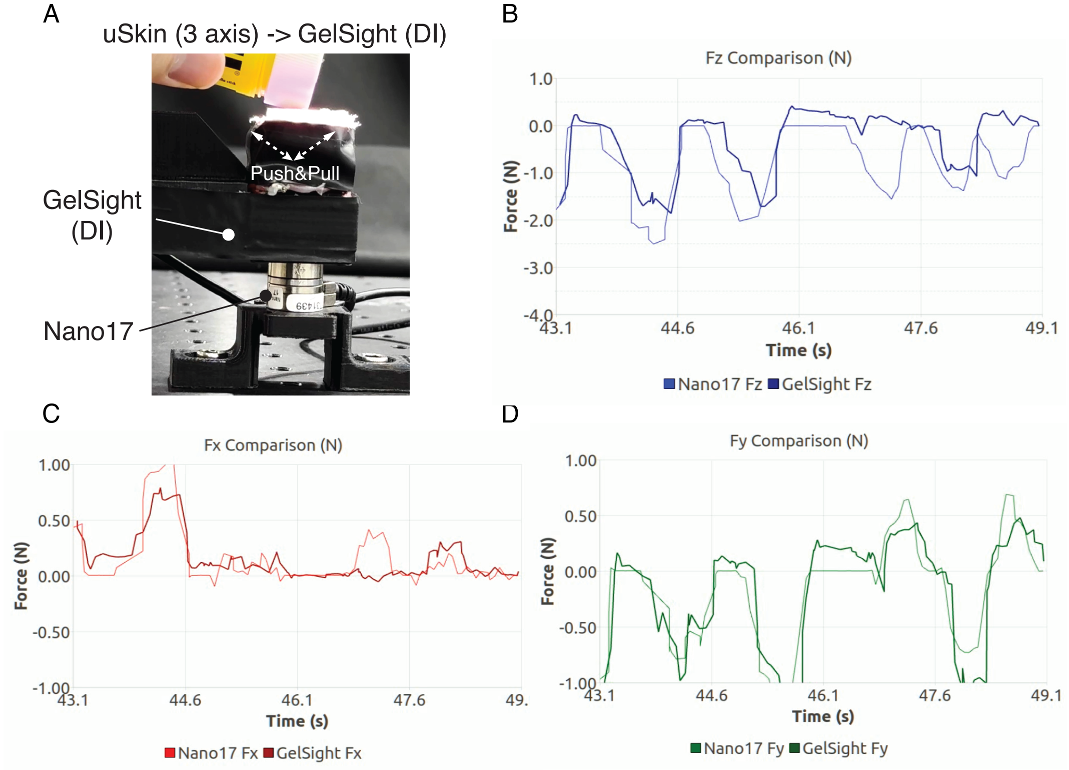

Supplementary Figure 24. Real-time force prediction when pushing and pulling on a GelSight (D-I) sensor. (A) Demonstration of test object, contact event, tactile sensor and nano17. The force model is transferred from a uSkin (3-axis) sensor. **(B-D)** Force prediction performance in Z-axis, X-axis and Y-axis respectively.

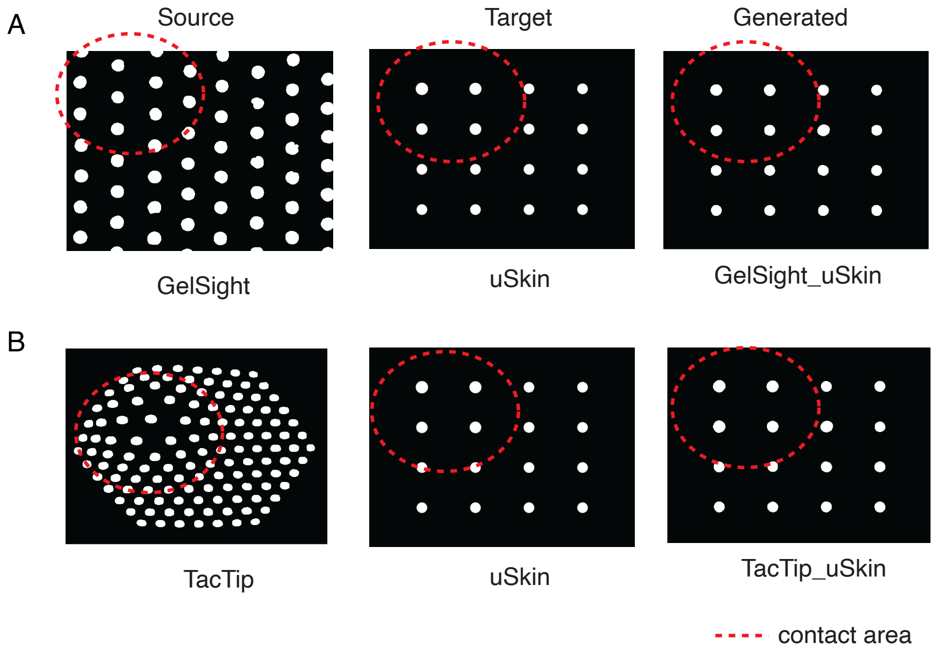

Supplementary Figure 25. Marker-to-marker result among GelSight (D-I), TacTip (palm) and uSkin with only z-axis component. (A) Transferring marker image from GelSight (D-I) to uSkin (z-axis). **(B)** Transferring from marker image TacTip (palm) to uSkin (z-axis). Note that, no shear displacement in uSkin's marker image due to only use signal from z-axis, similar to other type of sensor array only with z-axis (pressure) sensing capability.

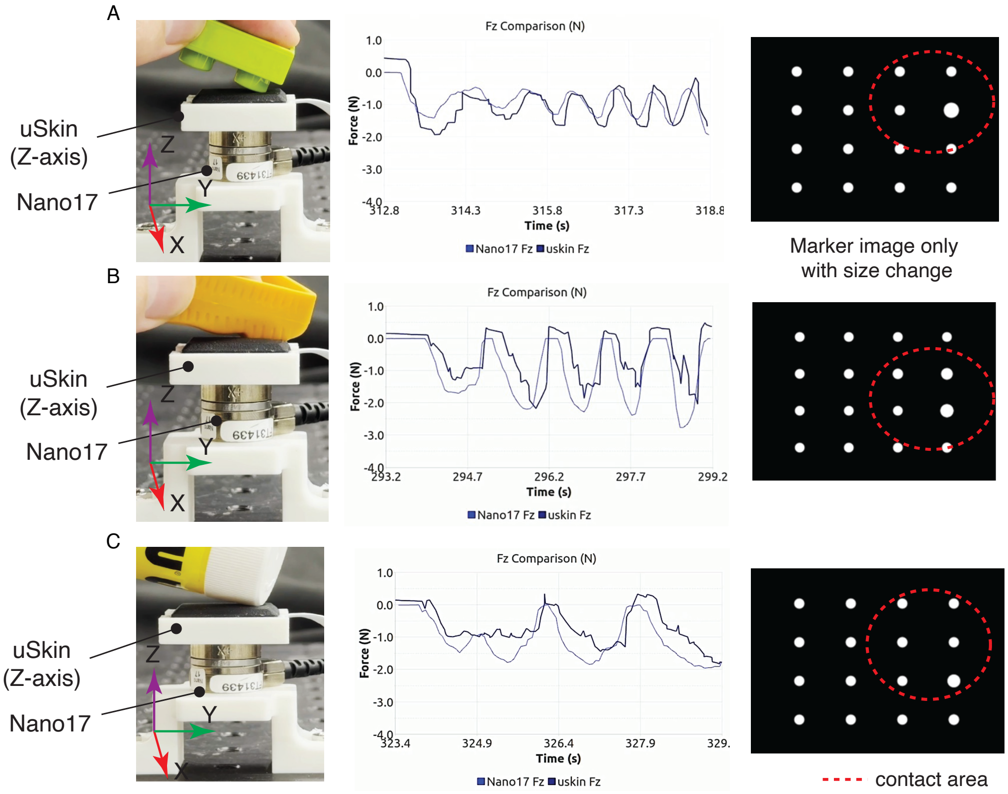

Supplementary Figure 26. Dynamic force test for uSkin (z-axis) with force model transferred from GelSight (D-I). (A) Continuous pressing with a Lego block. **(B)** Rubbing with a plastic Pizza from YCB. **(C)** Pushing with a glue stick. All test is compared with ATI nano 17. See more details in our Supplementary Video 3.

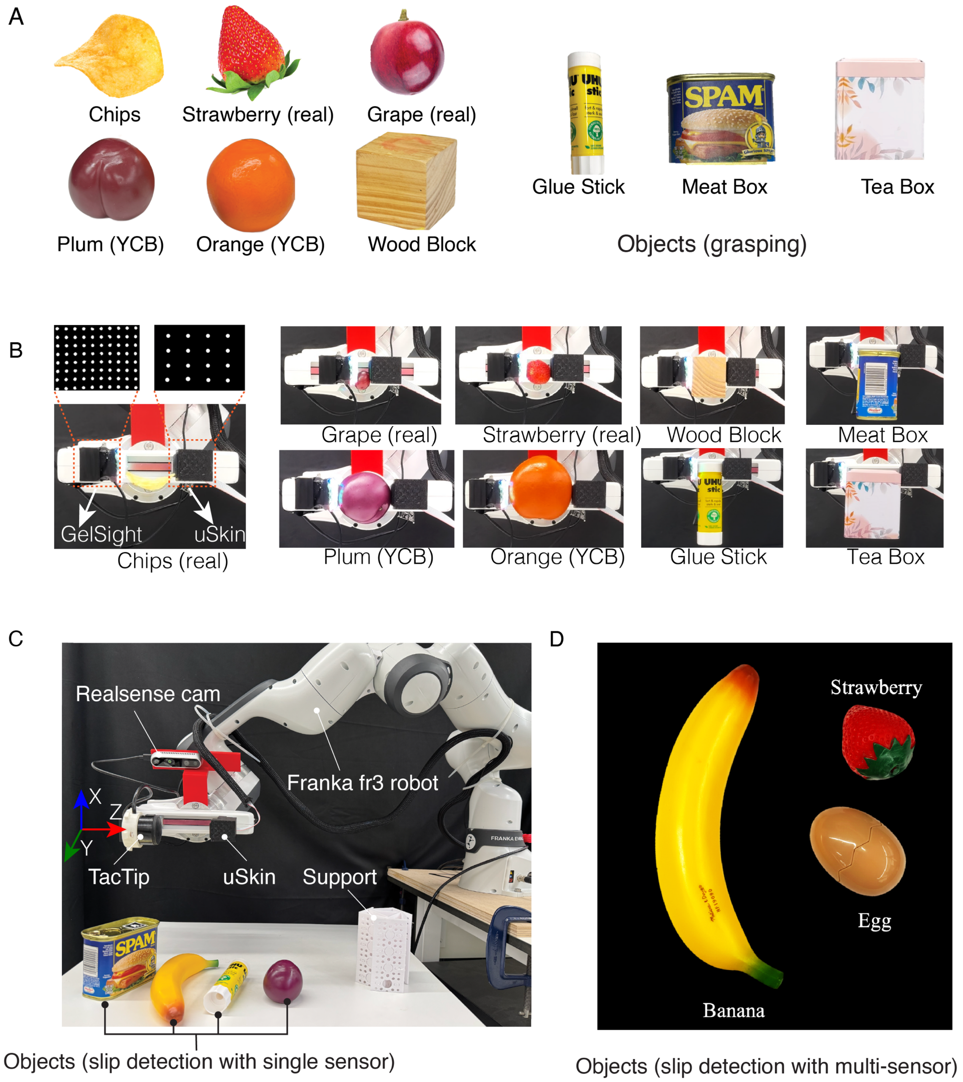

Supplementary Figure 27. Transferable force sensing in real-robot tasks. (A) Objects used for robot grasping task. **(B)** Daily objects grasping with transferable force sensing and control, using GelSight (A-II) and uSkin (three-axis). **(C)** Objects and setup used for robot slip detection and compensation with single sensor. **(D)** Objects and setup used for robot slip detection and compensation with multiple sensors.

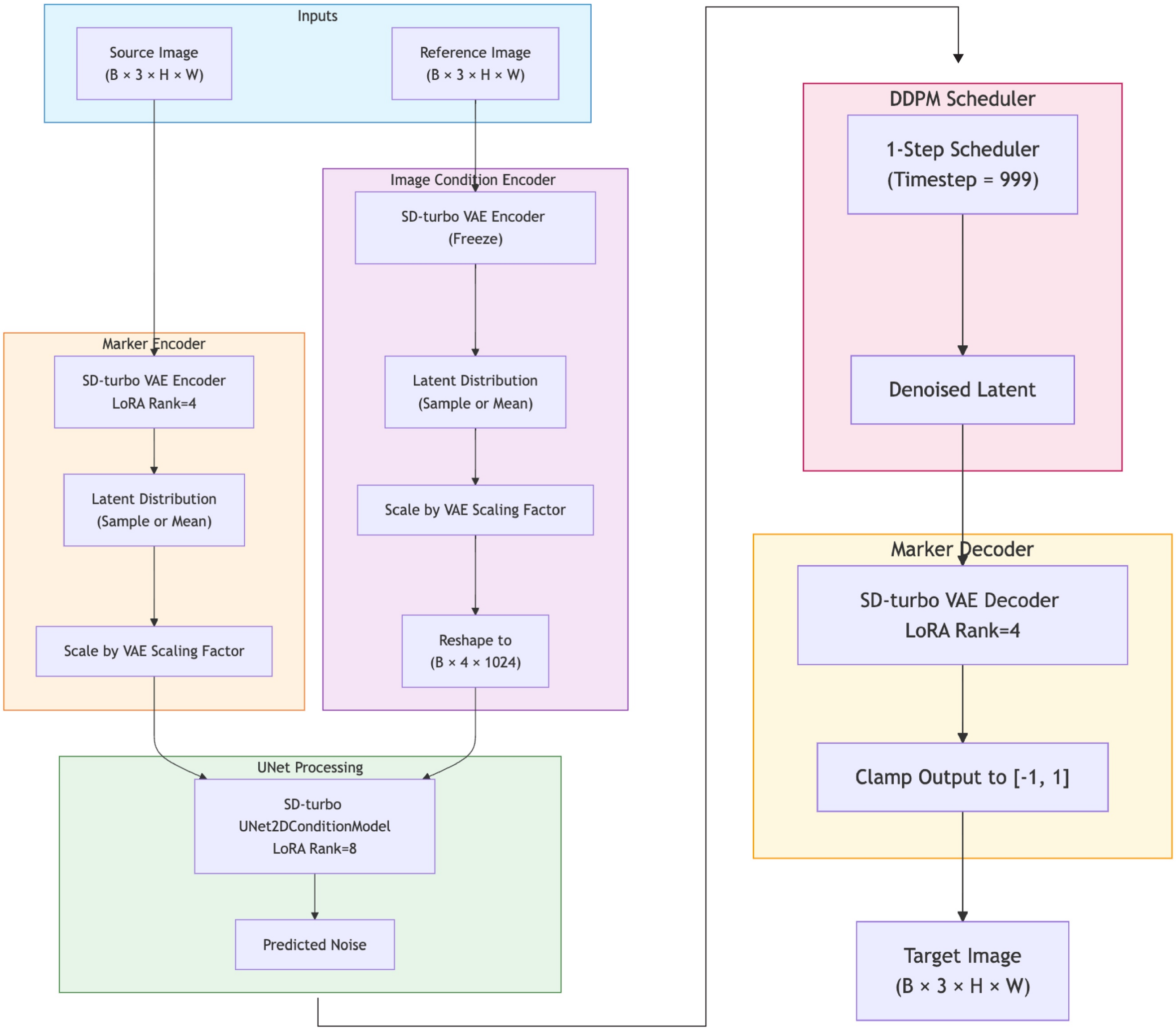

Supplementary Figure 28. Maker-to-marker translation model architecture.

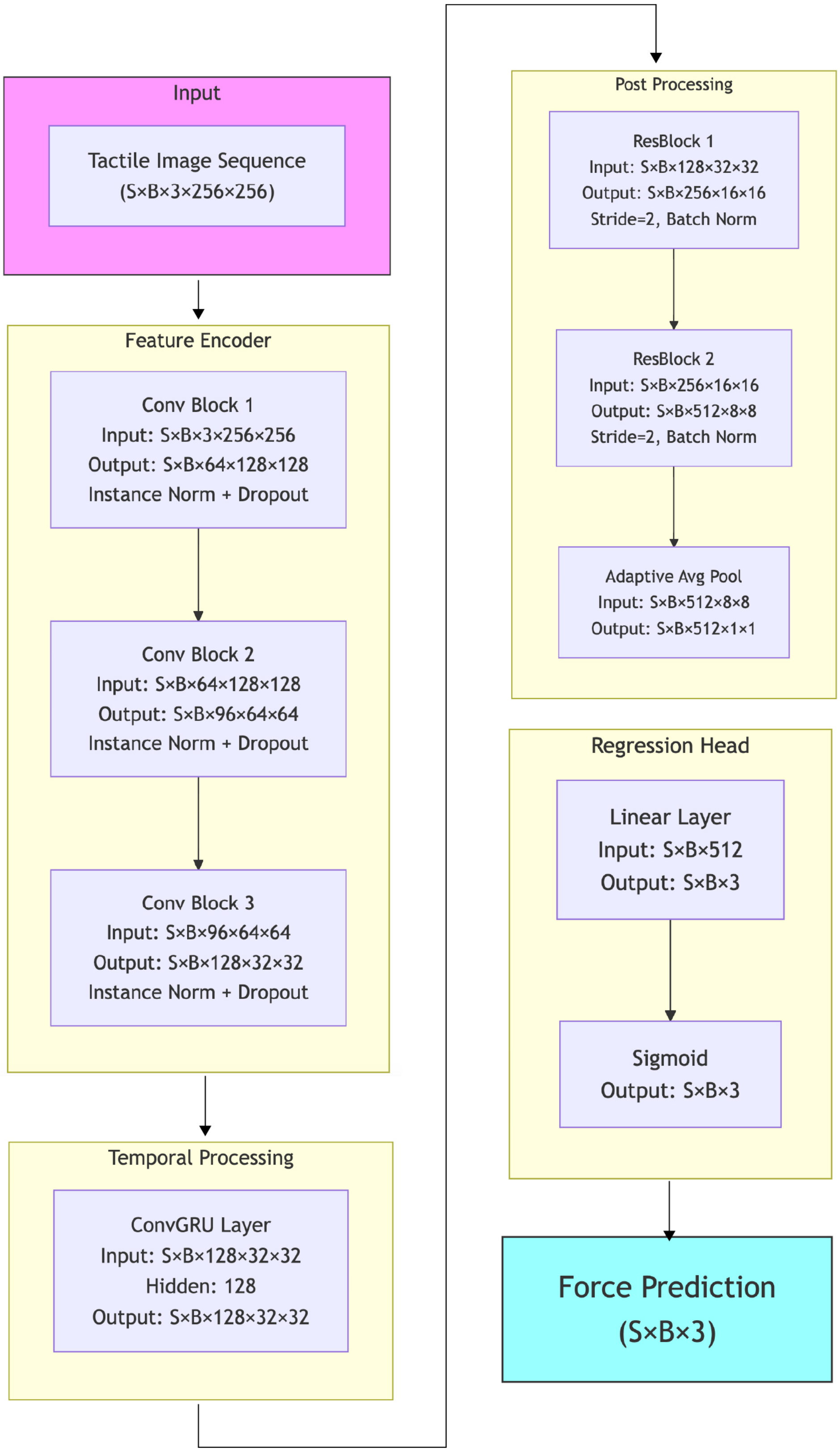

Supplementary Figure 29. Spatiotemporal force prediction model architecture.

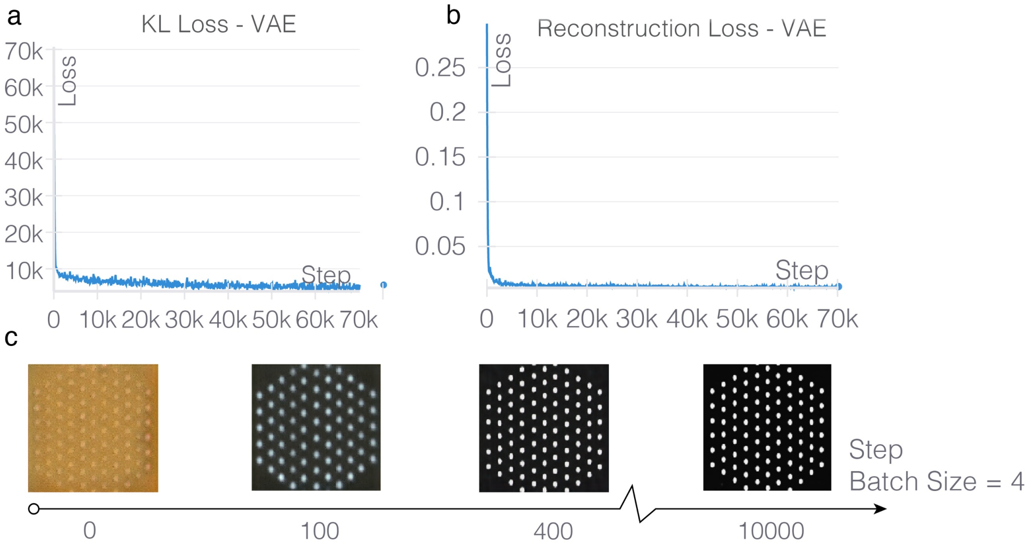

Supplementary Figure 30. Training process for the marker encoder-decoder. (A) KL Loss. **(B)** Reconstruction Loss. **(C)** The development process of decoded images.

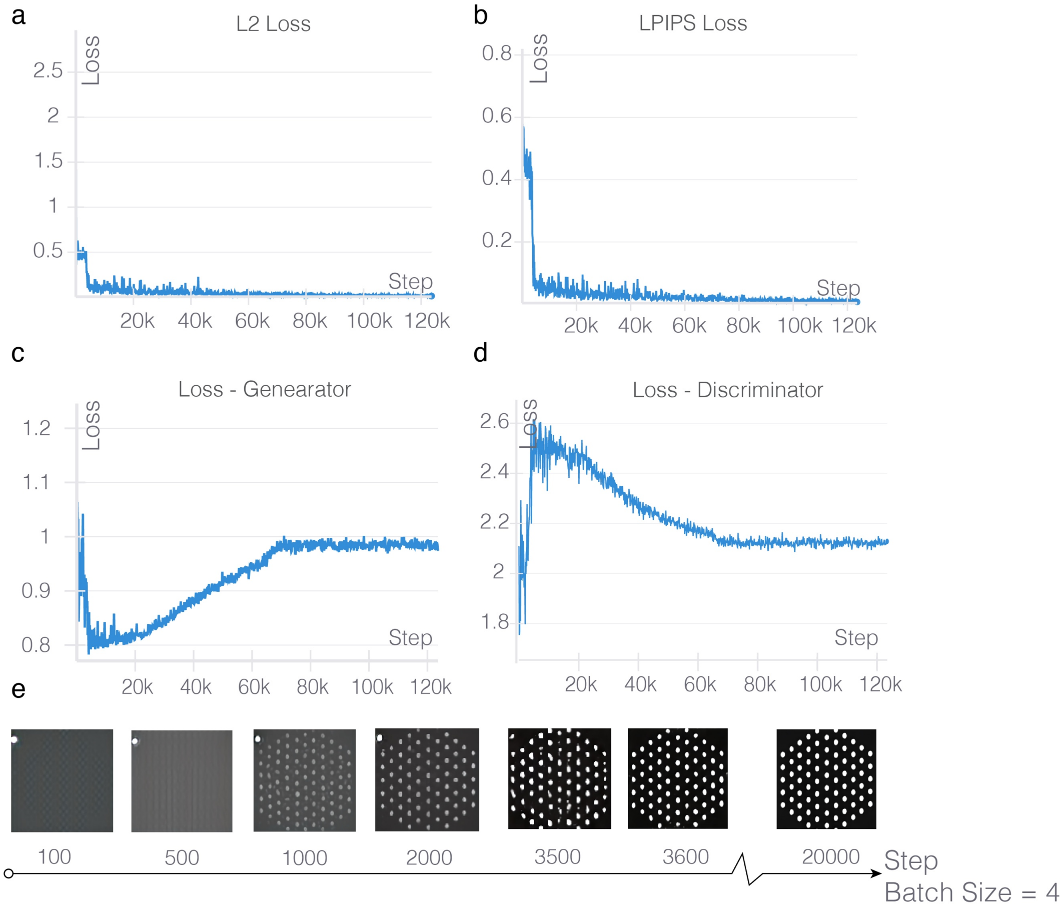

Supplementary Figure 31. Training Process for M2M model with simulated data. (A) L2 loss. **(B)** LPIPS loss. **(C)** Generator loss. **(D)** Discriminator loss. **(E)** The development process of generated images with simulated data.

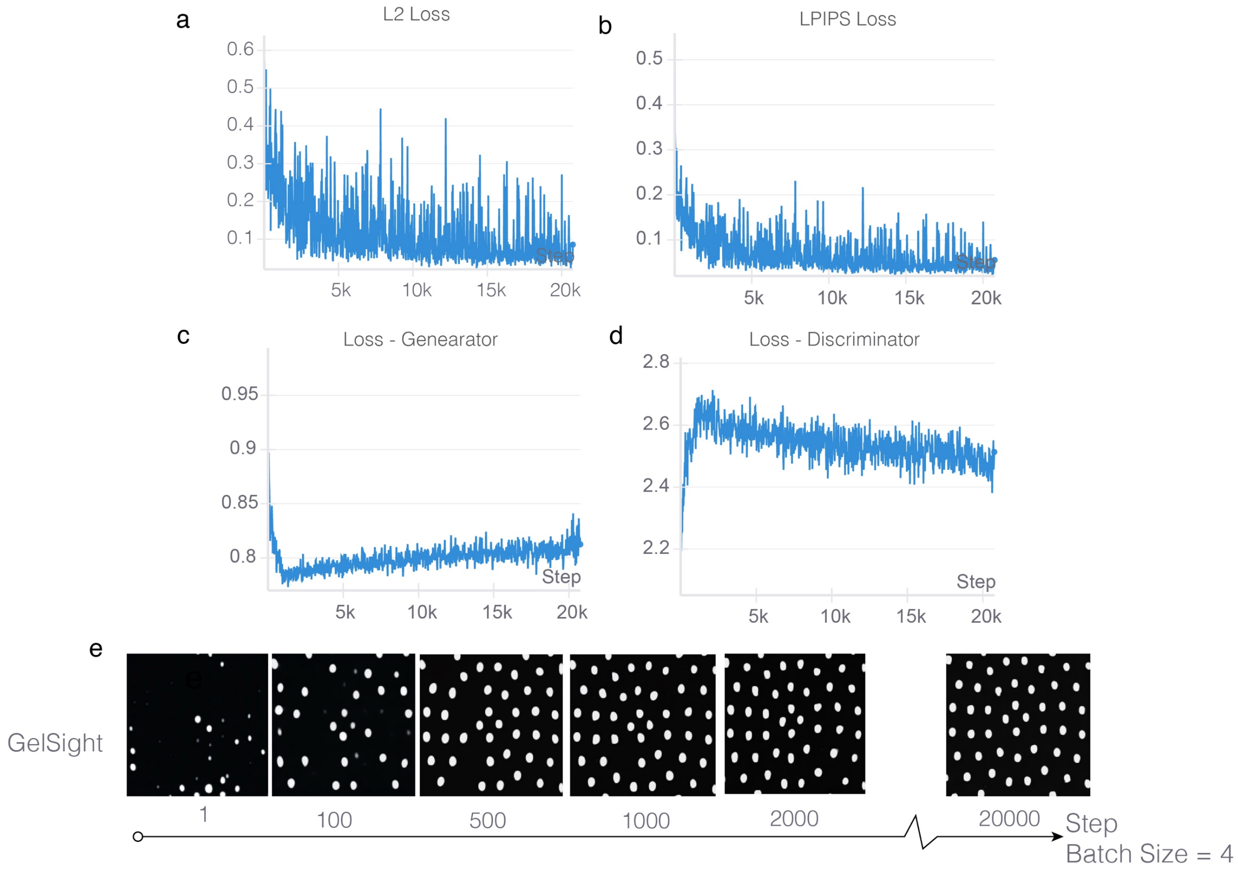

Supplementary Figure 32. Training Process for M2M model with homogeneous sensors. (A) L2 loss. **(B)** LPIPS loss. **(C)** Generator loss. **(D)** Discriminator loss. **(E)** The development process of generated images from homogeneous GelSight sensors.

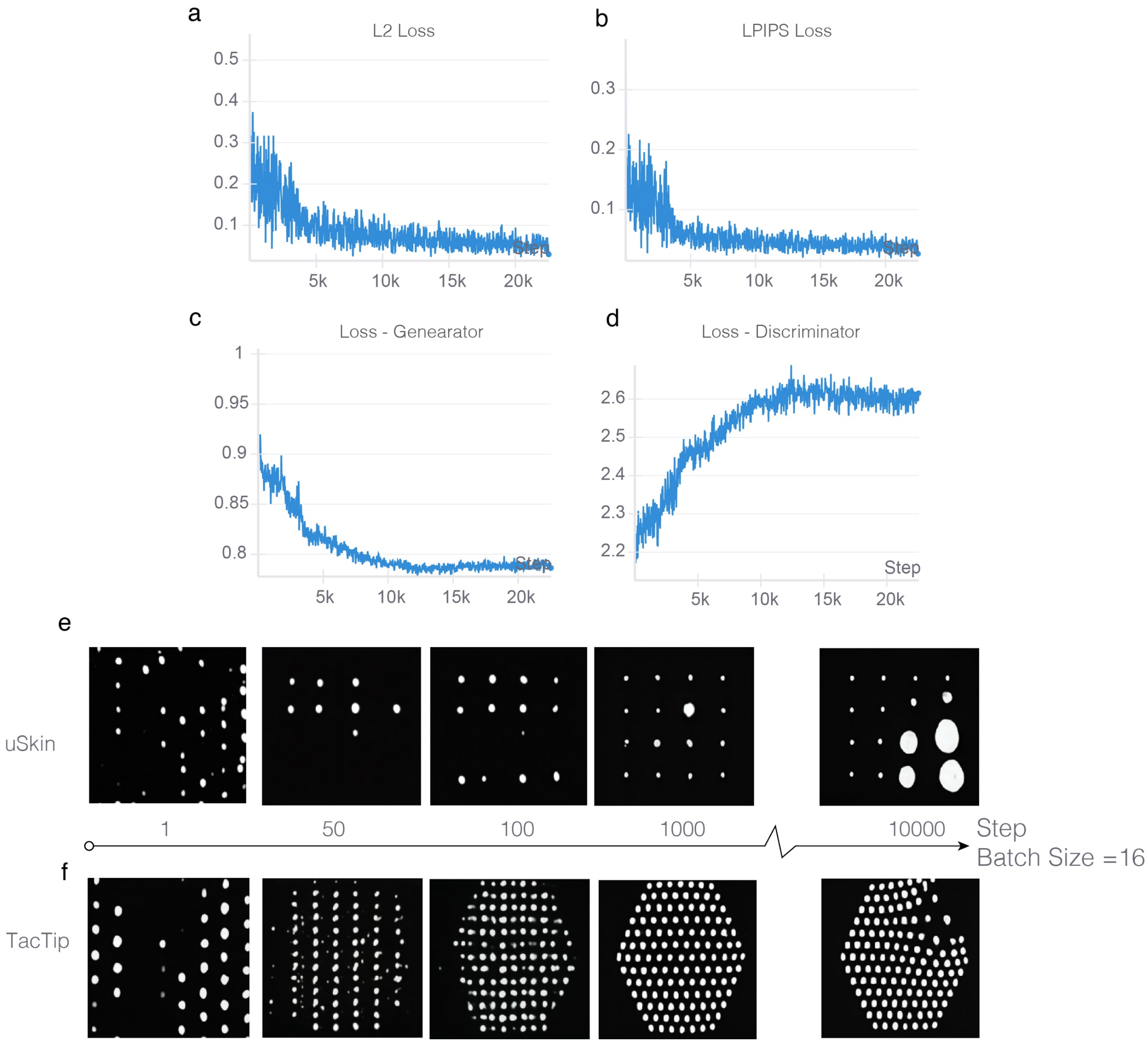

Supplementary Figure 33. Training Process for M2M model with heterogeneous sensors. (A) L2 loss. **(B)** LPIPS loss. **(C)** Generator loss. **(D)** Discriminator loss. **(E)** The development process of generated images from uSkin. **(F)** The development process of generated images from TacTip.

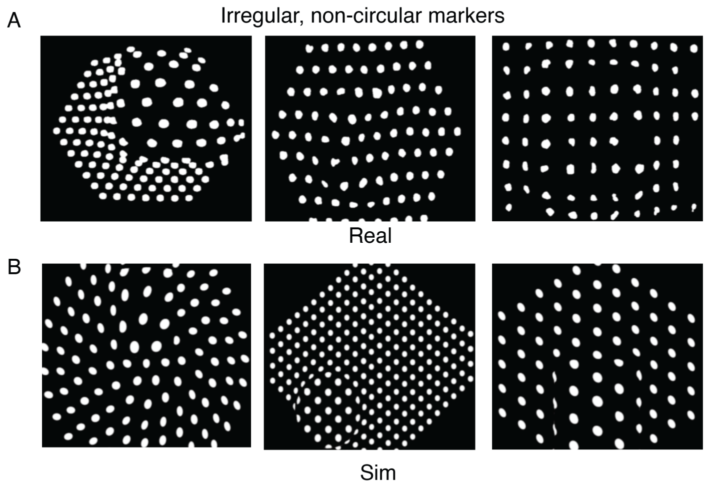

Supplementary Figure 34. Irregular and non-circular markers in our dataset. (**A**) Real-world marker images with explosive area, irregular edge and missing part. (**B**) Simulated marker images with elliptical shape, high-density and distortion.

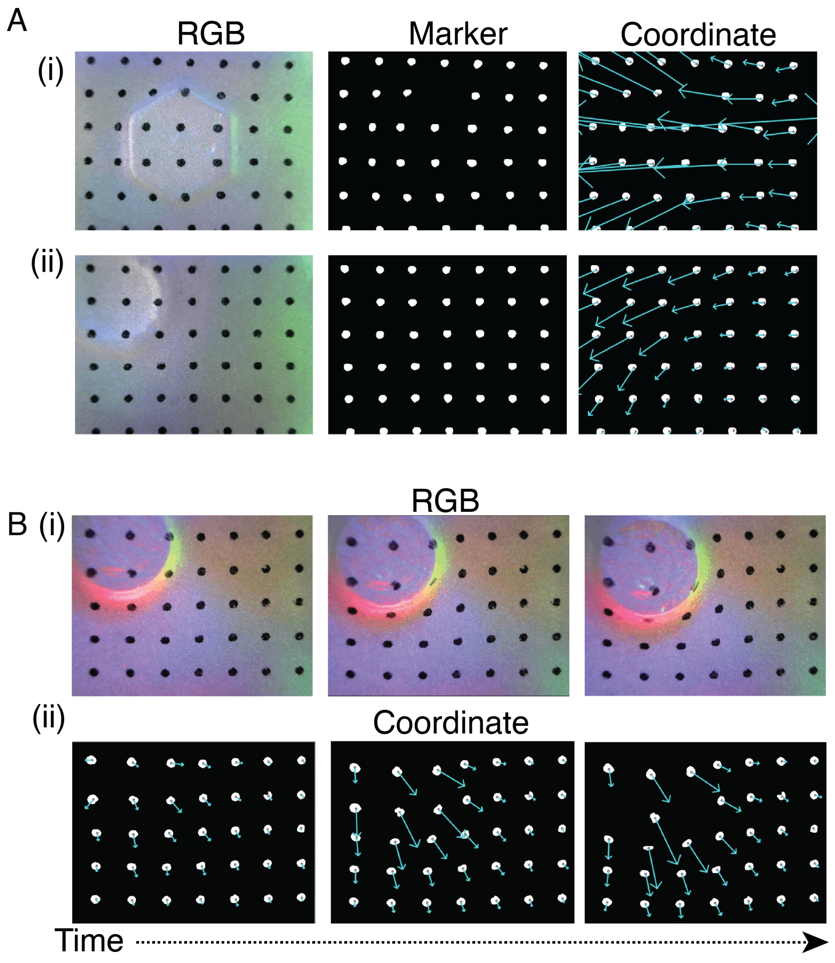

Supplementary Figure 35. Failure cases of marker coordinates. (**A**) Missing marker and losing tracking of marker coordinate (i) compared with normal tracking in static images. (**B**) Missing markers and losing track of marker coordinates in sequential images due to large displacement.

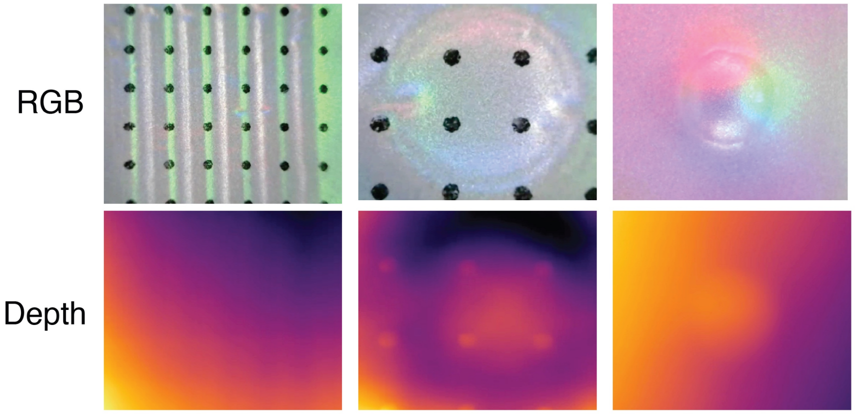

Supplementary Figure 36. Failure cases of depth image from vision-based tactile sensors. The depth images of contact area are hard to extract from RGB images using a Depth Anything model.

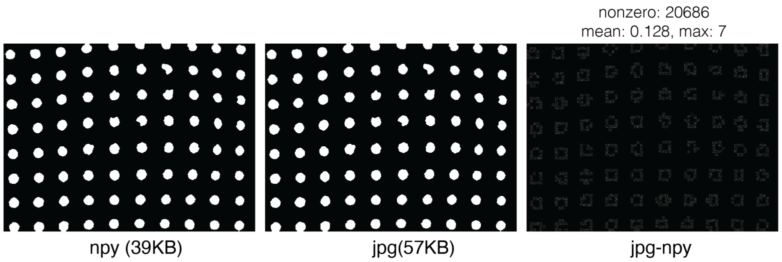

Supplementary Figure 37. Comparison of npy and jpg saving format for marker images. The npy saving format is uncompressed while only takes 39KB by packing into binary bit. However the jpg file takes 57KB and saves with edge pollution due to its compressed format.

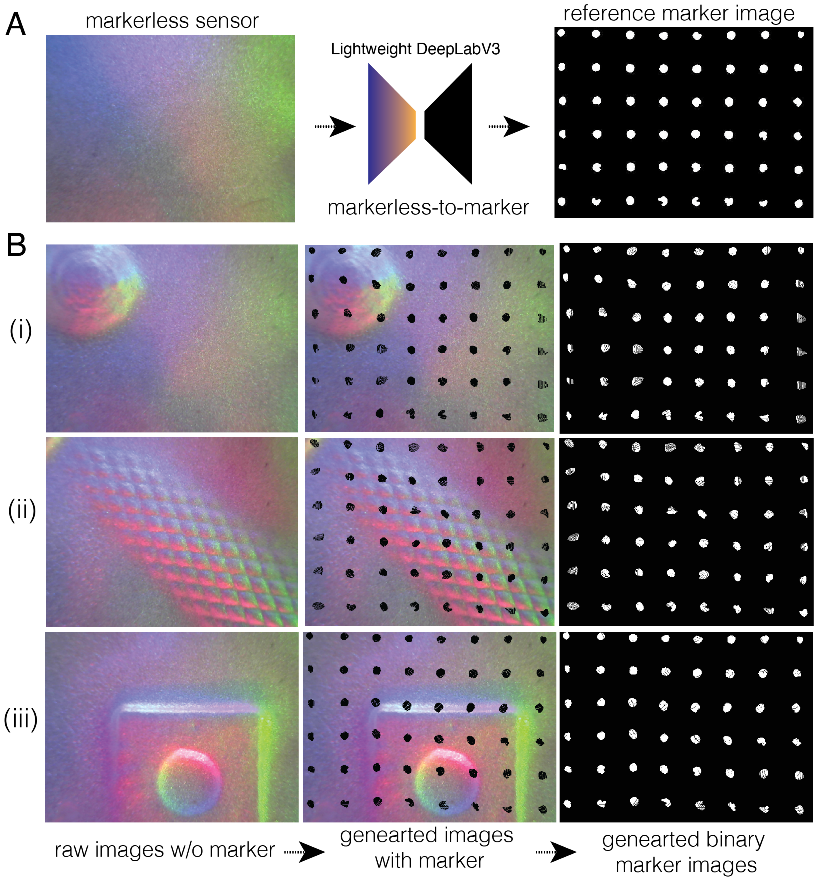

Supplementary Figure 38. Markerless-to-marker translation. (**A**) Pipeline for markerless to marker translation by using a lightweight real-time segmentation model DeepLabV3[52]. (**B**) Examples for translating contact image without (w/o) marker to binary marker images.

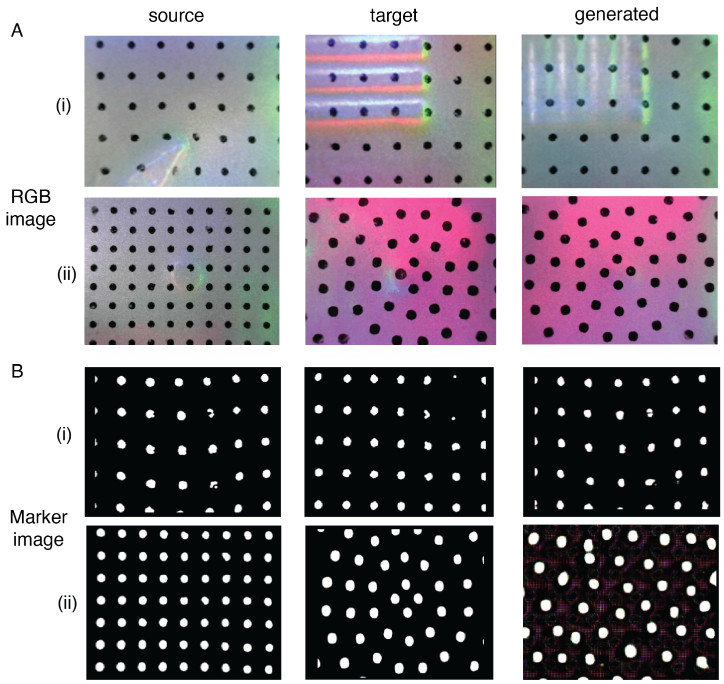

Supplementary Figure 39. Tactile image translation performance for using cycleGAN. (**A**) Failure case for transferring using RGB image. (i) Incorrect orientation of indenter. (ii) Incorrect circular marker pattern (**B**) Failure case for transferring using marker image. (i) Fail to transfer marker pattern with two grid-like marker images. (ii) Fail to converge between a grid marker image and circular marker image.

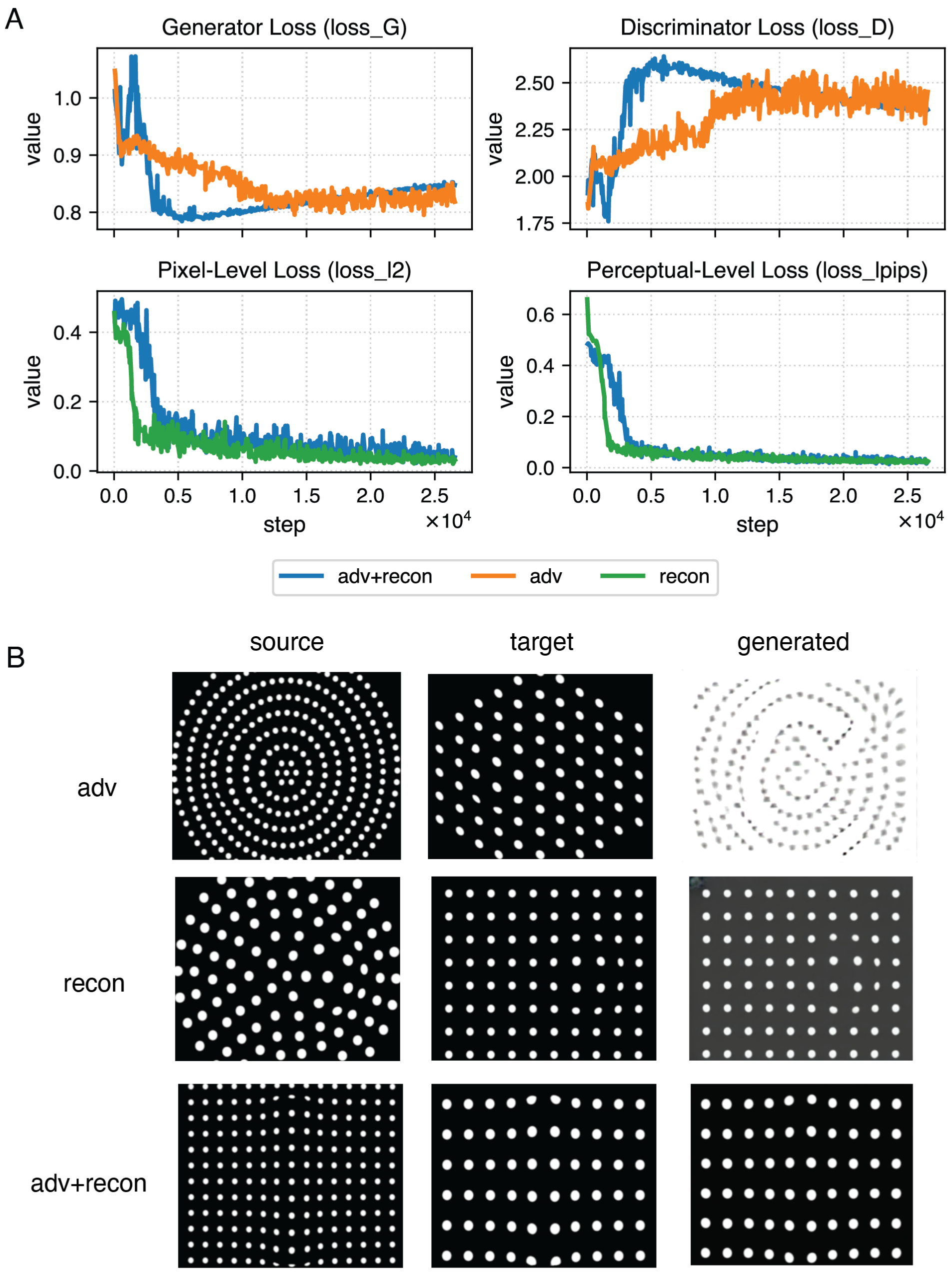

Supplementary Figure 40. Ablation study for the loss function in marker-to-marker translation model. (**A**) Loss curves for models with adversarial loss, reconstruction loss and both respectively. (**B**) Marker-to-marker translation results for three types of loss function.

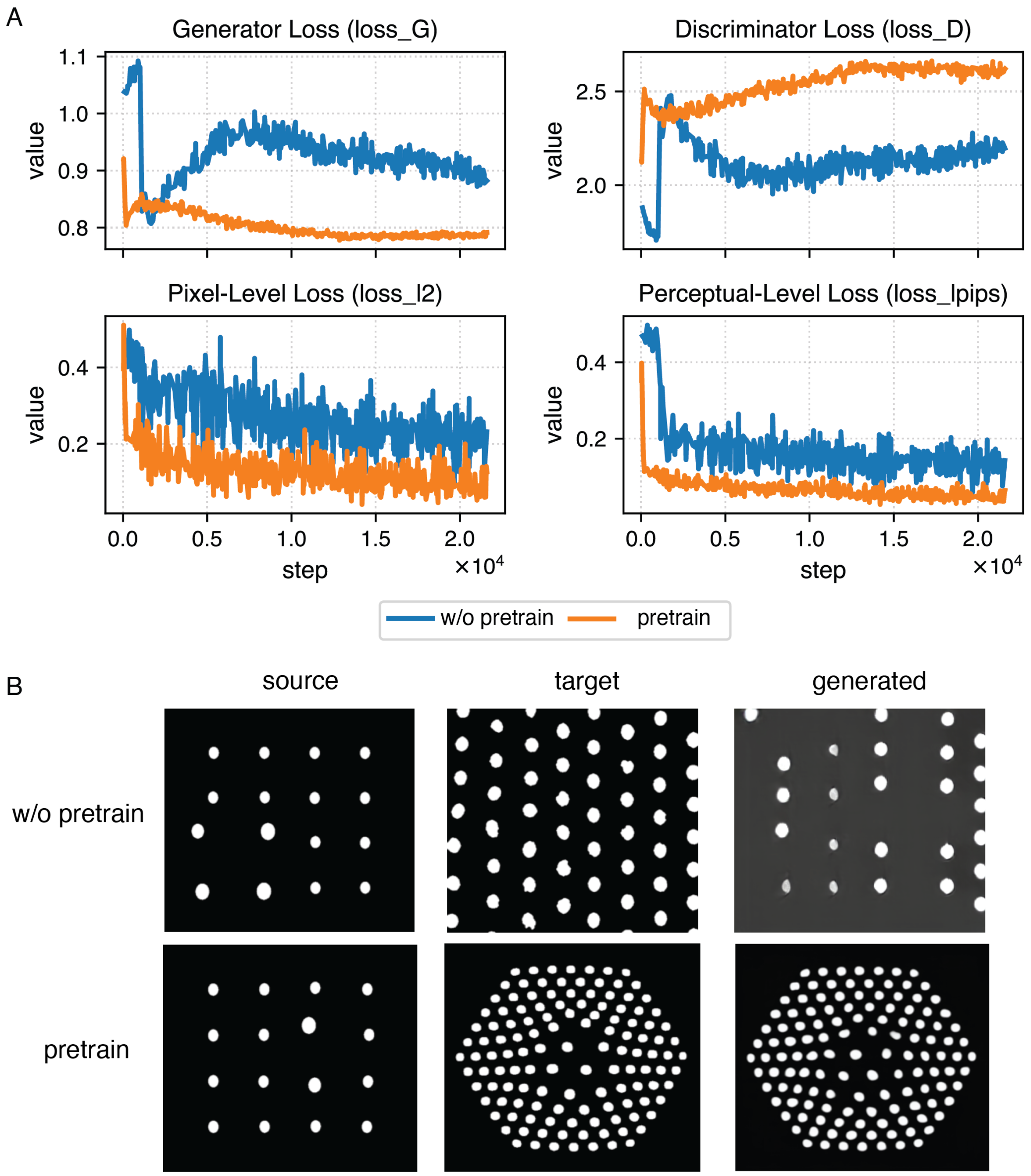

Supplementary Figure 41. Ablation study for the function of pretrained model with simulated data in marker-to-marker translation. (**A**) Loss curves for models with and without (w/o) pretraining respectively. (**B**) Marker-to-marker translation results for models with and without (w/o) pretraining.

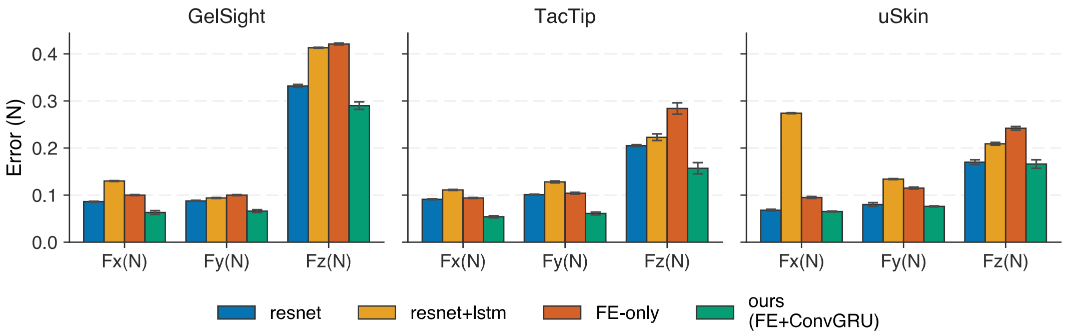

Supplementary Figure 42. Ablation study for force prediction performance of different modules trained with all indenters. We compare the force prediction in three axis when using ResNet, Resnet with LSTM, Feature Encoder-Only (FE-only) and our models (FE+ConvGRU) in three different sensors. Our model shows the lowest errors among all groups when training with 18 indenters in the training stage. We use random seed {0,10,20} to train 20 epochs with learning rate 0.1 plus another 20 epochs with learning rate 0.001. All error bars are ±1SD.

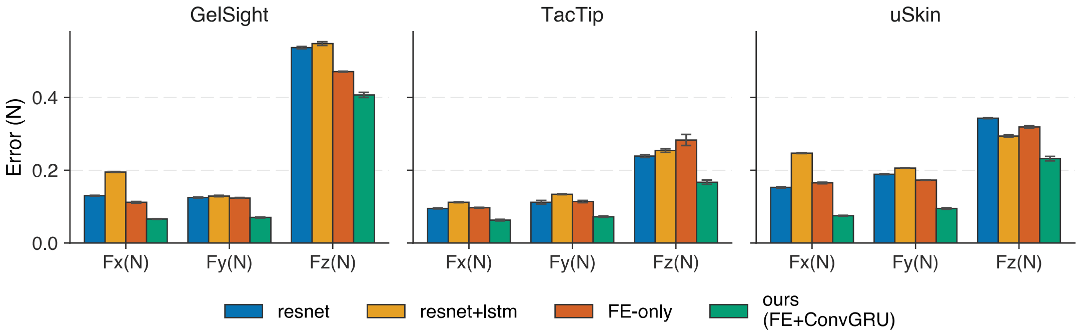

Supplementary Figure 43. Ablation study for force prediction performance of different modules tested with indenters unseen in training stage. We show force prediction error for 6 indenters unseen in training stage compared with ground truth from ATI nano17. Our model demonstrates the lowest errors among all groups. We use random seed {0,10,20} to train 20 epochs with learning rate 0.1 plus another 20 epochs with learning rate 0.001. All error bars are ±1SD.

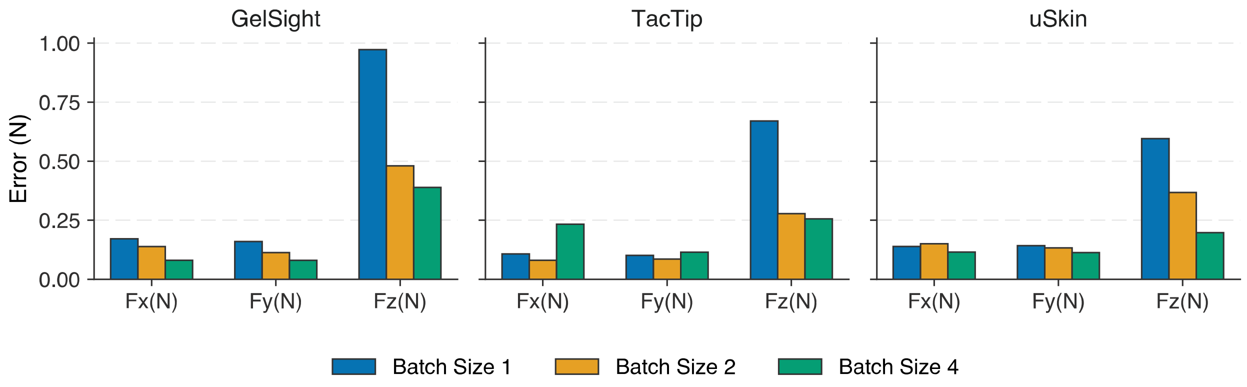

Supplementary Figure 44. Ablation study for batch size on force prediction performance. We show the force prediction errors for all indenters seen in training stage compared with ground truth from ATI nano17. When batch size is set to 1, the model performs poor in normal force. When batch size larger than 2, the model performs well. We use random seed 0, training 20 epochs with learning rate 0.1.

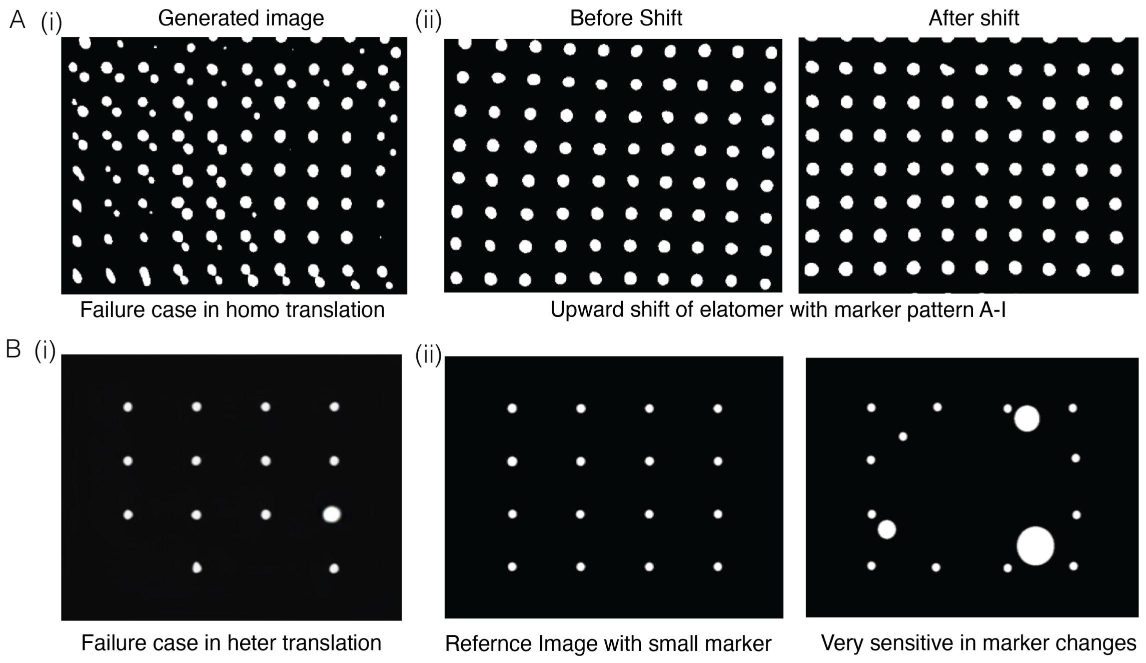

Supplementary Figure 45. Failure cases in M2M translation. (**A**) The generated image (i) transferring from GelSight (A-II) to GelSight (A-I) fails due to the shift (ii) of the elastomer, which disturbs image distribution of GelSight (A-I). (**B**) The M2M model is hard to converge (i) when the reference marker of uSkin (three-axis) is too small, or too sensitive in changes of marker size and marker displacement.

Supplementary Table 1. Ablation Study for the Number of Indenters in Seen Group on Force Prediction Performance

Number of Indenters (seen)	GelSight		
	Fx (N)	Fy(N)	Fz(N)
4	0.199	0.102	0.593
8	0.138	0.076	0.433
**12**	**0.066**	**0.069**	**0.399**

* Shown with force prediction error when tested in unseen group compared with ground truth from ATI nano17.
* We use our model (FE+convGRU) in this test.
* Random seed 0, training 20 epochs with learning rate 0.1.

Supplementary Table 2. Hyperparameters used in material compensation in study of material hardness effect

Source \ Target	r6	r8	r10	r12	r14	r16	r18
**r6**		0/0.5	0.4/1	0/0.5	0/0.75	0/0.75	0/0.75
**r8**	0/0		0.8/1	0/0.75	0/0.75	0/0.75	0/0.75
**r10**	0/0.5	0/0.75		0.4/0.25	0/0.5	0/0.5	0/0.5
**r12**	0.8/0.75	0.8/0.25	0.8/0.25		0/0.75	0/0.75	0/0.5
**r14**	0.8/0.5	0/0	0.8/0.25	0/0		0/0	0/0.75
**r16**	0.8/0.5	0/0	0/0	0/0	0/0		0/0.5
**r18**	0.4/0.25	0/0	0/0	0.8/0.5	0.8/0.25	0.4/0.25	

*Demonstrate starting depth d_0 (mm) and correction weights λ as d_0 / λ in each cell

*Grid search in range of [0,1] with a step of 0.4 for d_0 and 0.25 for λ

Supplementary Table 3. Hyperparameters used in material compensation in study of heterogeneous translation

Source \ Target	uSkin	GelSight	TacTip
uSkin		0.5/1	0/0.5
GelSight	0/0		0/0.75
TacTip	0.75/0.5	0/0.5	

*Demonstrate starting depth d_0 (mm) and correction weights λ as d_0 / λ in each cell

*Grid search in range of [0,1] with a step of 0.25 for d_0 and 0.25 for λ

Supplementary References

1. Yuan, W., Dong, S. & Adelson, E. GelSight: High-Resolution Robot Tactile Sensors for Estimating Geometry and Force. *Sensors* **17**, 2762 (2017).
2. Parmar, G., Park, T., Narasimhan, S. & Zhu, J.-Y. One-Step Image Translation with Text-to-Image Models. Preprint at https://arxiv.org/abs/2403.12036 (2024).
3. Goodfellow, I. J. et al. Generative Adversarial Networks. in *Advances in Neural Information Processing Systems* (2014).
4. Zhang, R., Isola, P., Efros, A. A., Shechtman, E. & Wang, O. The Unreasonable Effectiveness of Deep Features as a Perceptual Metric. in *2018 IEEE/CVF Conference on Computer Vision and Pattern Recognition (CVPR)* 586–595 (IEEE, 2018).
5. Sauer, A., Lorenz, D., Blattmann, A. & Rombach, R. Adversarial Diffusion Distillation. in *The European Conference on Computer Vision* 87–103 (IEEE, 2024).
6. Hu, E. J. *et al.* LoRA: Low-Rank Adaptation of Large Language Models. *The Tenth International Conference on Learning Representations* (2021).
7. Ronneberger, O., Fischer, P. & Brox, T. U-Net: Convolutional Networks for Biomedical Image Segmentation. in *Medical Image Computing and Computer-Assisted Intervention* 234–241 (2015).
8. Ho, J., Jain, A. & Abbeel, P. Denoising Diffusion Probabilistic Models. in *the 34th International Conference on Neural Information Processing Systems* 6840–6851 (2020).
9. Kumari, N., Zhang, R., Shechtman, E. & Zhu, J.-Y. Ensembling Off-the-shelf Models for GAN Training. in *Proceedings of the IEEE/CVF conference on computer vision and pattern recognition (CVPR)* 10651-10662 (2022).
10. Teed, Z. & Deng, J. RAFT: Recurrent All-Pairs Field Transforms for Optical Flow. in *European Conference on Computer Vision (ECCV)* 402–419 (2020).
11. Zhu, J. Y., Park, T., Isola, P., & Efros, A. A. Unpaired image-to-image translation using cycle-consistent adversarial networks. In *Proceedings of the IEEE international conference on computer vision (CVPR)* 2223-2232 (2017).

12. Fischer-Cripps, A. C. *Introduction to Contact Mechanics* (Springer New York, NY, 2010). https://doi.org/10.1007/978-0-387-68188-7.
13. Popov, V. L., Heß, M., & Willert, E. *Handbook of contact mechanics: exact solutions of axisymmetric contact problems* (Springer Berlin, Heidelberg, 2019).
14. Zhao, C., Ren, J., Yu, H. & Ma, D. In-situ Mechanical Calibration for Vision-based Tactile Sensors. in *2023 IEEE International Conference on Robotics and Automation (ICRA)* 10387–10393 (2023).
15. Li, M., Zhang, L., Zhou, Y. H., Li, T. & Jiang, Y. EasyCalib: Simple and Low-Cost In-Situ Calibration for Force Reconstruction With Vision-Based Tactile Sensors. *IEEE Robot Autom Lett* **9**, 7803–7810 (2024).
16. Yuan, W., Li, R., Srinivasan, M. A., & Adelson, E. H. Measurement of shear and slip with a GelSight tactile sensor. In *2015 IEEE International Conference on Robotics and Automation (ICRA)* 304-311 (IEEE, 2015).
