# Supplementary material for: Training tactile sensors to learn force sensing from each other
Source: Nat Commun. 2026 Jan 28;17:2101. doi: 10.1038/s41467-026-68753-1 (PMC12953886; doi:10.1038/s41467-026-68753-1)
Supplement: Supplementary file 2 — Description of Additional Supplementary Information [file 41467_2026_68753_MOESM2_ESM.pdf]

Description of Additional Supplementary Files

File Name: Supplementary Video 1
Description: Marker-to-marker translation.

File Name: Supplementary Video 2
Description: Force prediction performance tested with ATI nano17.

File Name: Supplementary Video 3
Description: Dynamic force test compared with ATI nano17.

File Name: Supplementary Video 4
Description: Daily objects grasping with heterogeneous sensors (1).

File Name: Supplementary Video 5
Description: Daily objects grasping with heterogeneous sensors (2).

File Name: Supplementary Video 6
Description: Slip detection and compensation with single-sensor control.

File Name: Supplementary Video 7
Description: Slip detection and compensation with multi-sensor coordination.
